# Patterns of divergence in the morphology of ceratopsian dinosaurs: sympatry is not a driver of ornament evolution

**DOI:** 10.1098/rspb.2018.0312

**Published:** 2018-03-21

**Authors:** Andrew Knapp, Robert J. Knell, Andrew A. Farke, Mark A. Loewen, David W. E. Hone

**Affiliations:** 1 Queen Mary University of London, London, UK; 2 Raymond M. Alf Museum of Paleontology, Claremont, CA, USA; 3 Natural History Museum of Utah, Salt Lake City, UT, USA

**Keywords:** evolution, sympatry, Dinosauria, Ceratopsia, species recognition, ornamentation

## Abstract

Establishing the origin and function of unusual traits in fossil taxa provides a crucial tool in understanding macroevolutionary patterns over long periods of time. Ceratopsian dinosaurs are known for their exaggerated and often elaborate horns and frills, which vary considerably between species. Many explanations have been proposed for the origin and evolution of these ‘ornamental’ traits, from predator defence to socio-sexual dominance signalling and, more recently, species recognition. A key prediction of the species recognition hypothesis is that two or more species possessing divergent ornamental traits should have been at least partially sympatric. For the first time to our knowledge, we test this hypothesis in ceratopsians by conducting a comparison of the morphological characters of 46 species. A total of 350 ceratopsian cladistic characters were categorized as either ‘internal’, ‘display’ (i.e. ornamental) or ‘non display’. Patterns of diversity of these characters were evaluated across 1035 unique species pairs. Display characters were found to diverge rapidly overall, but sympatric species were not found to differ significantly in their ornamental disparity from non-sympatric species, regardless of phylogenetic distance. The prediction of the species recognition hypothesis, and thus the idea that ornamentation evolved as a species recognition mechanism, has no statistical support among known ceratopsians.

## 1. Introduction

Exaggerated and elaborate anatomical features are well known among many fossil taxa, including trilobites, amphibians, non-avian dinosaurs and artiodactyls [1]. These features can take the form of bony processes, spines, horns, crests and frills, often with no obvious functional explanation. A number of hypotheses have been suggested for the presence and evolution of such ornaments, including predator defence, mechanical support, thermoregulation, social or sexual signalling, and species recognition [2–5]. The first three of these proposed explanations have been considered and ruled out in many analyses of specific systems, leaving species recognition and sexual selection as the two main competing hypotheses that could offer a more general explanation for the evolution of ornaments. There is now growing evidence that sexual selection can influence macroevolutionary processes such as speciation, extinction and adaptation. The fossil record offers the opportunity to test these ideas over considerably longer timescales than possible in extant organisms.

**Figure 1.**
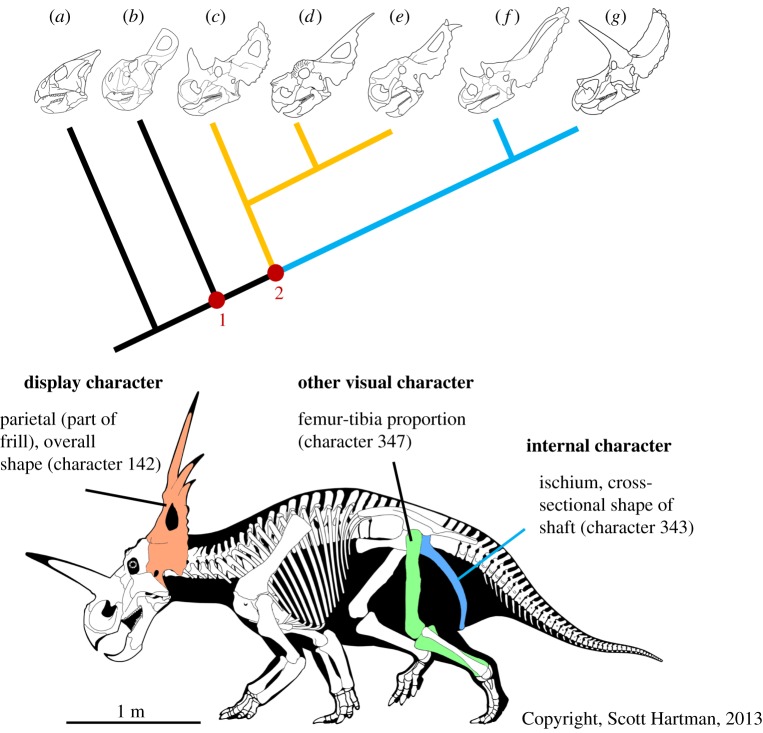
Line drawings of ceratopsian skulls in simplified phylogeny to illustrate morphological diversity of cranial ornaments within the clade. (*a*) *Liaoceratops yangzigouensis*; (*b*) *Protoceratops andrewsi*; (*c*) *Centrosaurus apertus*; (*d*) *Achelousaurus horneri*; (*e*) *Pachyrhinosaurus canadensis*; (*f*) *Chasmosaurus belli*; (*g*) *Triceratops horridus*. Node 1 represents the clade Coronosauria, containing all taxa with enlarged frills. Node 2 represents the clade Ceratopsoidea, encompassing Centrosaurinae (orange branch) and Chasmosaurinae (blue branch), within which the majority of cranial ornamental diversity, and all horned taxa, are found. Lower image: full-body illustration of *Styracosaurus albertensis* (Centrosaurinae) with highlighted examples of the three different character classes used in this study (refer to the electronic supplementary material for full list of characters).

The Ceratopsia was a major clade of non-avialan dinosaurs (hereafter ‘dinosaurs’) of over 70 known species, all of which possessed large, ornamented, morphologically diverse skulls [6,7]. (‘Ornament’ refers here to any exaggerated morphological feature that was externally visible in life and has no obvious utilitarian function, presumably functioning, in part, as a visual signal. We do not distinguish between ornaments and weapons to avoid presupposing function [8].) Ceratopsians are well represented in the fossil record and, coupled with their diverse skull morphology and rapid species turnover, are well-suited for the study of macroevolutionary patterns. Cranial ornamentation in ceratopsians variously takes the form of frills (composed of enlarged parietal and squamosal bones), nasal and postorbital horns, prominent jugal (‘cheek’) spikes, and epiossifications around the posterior margin of the frill (figure 1) [9]. Sexual dimorphism is not known from any ceratopsian species [10], and no similar ornamentation is found in extant taxa. Early ceratopsians, such as *Psittacosaurus* and *Liaoceratops* (figure 1*a*), had combinations of enlarged jugal spikes, and small, incipient frills [6]. Enlarged frills and horns appeared first in the basal neoceratopsian *Zuniceratops* [11], and reached their peak diversity and complexity in the Centrosaurinae between 80 and 70 Ma (figure 1) [7,12]. Despite the great diversity in ornament morphology, at no point were horns or frills completely lost in any ceratopsian lineage once established.

Sexual selection is difficult to determine in extinct taxa because of the lack of behavioural or genetic data, and there is no single, reliable means of recognizing it from morphology alone [1]. The absence of any obvious sexual dimorphism has led some to conclude that sexual selection cannot provide an explanation for ornamentation in ceratopsians, and that the best explanation is, therefore, species recognition [13]. Species recognition has been proposed as an alternative hypothesis for the divergence of ornaments in extant species as a means of avoiding hybridization; closely-related sympatric species should, it is argued, develop contrasting morphological characters to differentiate themselves, a process known as character displacement [14–18]. Such characters are not expected to have an obvious mechanical function, and the process is distinct from ecological character displacement [19]. Morphological characters involved in species recognition are expected to function early on in social interactions between individuals [20] and should be highly visible, an obvious characteristic of highly ornamented ceratopsian skulls.

Padian and Horner suggested the following two tests for identifying species recognition traits in extinct animals [3]:

(i) patterns of diversification of ornaments should be relatively random; and
(ii) there should be evidence that at some point, several closely related species with divergent ornaments lived at the same time in environments that at least partially overlap.

The first hypothesis is not useful in distinguishing species recognition traits because sexually selected features also sometimes show apparently ‘random’ patterns of divergence [21–23]. Given the reasonably dense fossil record of the Ceratopsia, however, it should be possible to test a modified version of the second hypothesis using species that are known to have been sympatric. If species recognition is a driver of trait evolution, then morphological disparity in ornamentation should be significantly higher between sympatric species because species that are not sympatric have minimal selective pressure to evolve obvious visual differences [15]. Species recognition should, therefore, promote the divergence of these traits to a greater degree between sympatric species than between species which did not coexist. The species recognition hypothesis can be easily tested with knowledge of morphology and species distributions, both of which are well known in ceratopsians.

Here, we perform, to our knowledge, the first ever evaluation of species recognition in a fossil vertebrate clade with an assessment of ceratopsian dinosaur data. We combine information on ornamental and other traits with information on sympatry to test the hypothesis that sympatric ceratopsian taxa evolved features that visually distinguish them from one another.

## 2. Methods

All known valid ceratopsian species were listed using existing sources [12,24]. Known geographical occurrences for each species were determined from the primary literature (electronic supplementary material, table S1). Temporal ranges follow published dates of their host formations, using more precise dates for occurrences within the formation where possible.

Phylogenetic information was compiled from a number of sources, and a composite tree constructed (electronic supplementary material) [7,9,25–36]. The phylogenetic distance between each species pair was calculated using a method [37] adapted for extinct taxa; the number of speciation events since the last common ancestor was counted for each possible species pair, omitting species radiations that occurred after the more recent occurrence of the two species. This avoids overweighting of clades that subsequently underwent large species radiations.

Trait comparisons were performed using a total of 350 defined and scored cladistic characters (electronic supplementary material). Each character was classed as either *external* or *internal,* based on whether it was likely to have an effect on the exterior appearance of the animal in life. External characters were further subdivided into *display* and *non-display*, defined as whether or not the character in question was deemed whole or part of an ornament (i.e. in ceratopsians, the frill, horns and bosses of the skull). The classification of characters in this way resulted in three character classes:

 internal characters (196 characters),
 display characters (86 characters),
 non-display (other) characters (68 characters).

Each species pair was compared using the following formula:
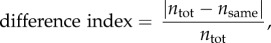
where *n*_tot_ is the total number of characters present in both taxa and *n*_same_ is the number of characters with matching states in both taxa. This resulted in a ‘difference index’ value of between 0 and 1, where species pairs possessing all identical characters are scored 0 and absolutely different pairs are scored 1.

Because of the low confidence arising from comparisons of poorly known species with many unknown characters, a minimum cut-off of 40% possession of characters was used. This retained a high number of species in the analysis while simultaneously excluding those known from limited remains. This latter group contained mainly the earlier, more basal members of the clade, and was deemed an acceptable omission because this study is concerned with the exaggerated structures associated primarily with Neoceratopsia. After these criteria were applied, a total of 46 ceratopsian species were retained from the initial total of 77. Phylogenetic distance values were not altered.

Comparisons of species pairs were determined by a pairwise comparison grid of 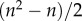 possible combinations, where *n* is the number of taxa. If we define sympatry as the situation where species pairs are known to be found in the same place and at the same time, we classify only 38 species pairs of a total of 1035 used in this study as sympatric. Given the incomplete nature of the fossil record and the possibility of dating errors, however, this is likely to be an underestimate, so we used a series of criteria to describe how likely each species pair was to have been present in the same place at the same time, as follows:

— *sympatric*: species pairs are found in the same location with overlap in their temporal range (38 pairs);
— *allopatric*: species pairs overlap in their temporal range and are found on the same continent, but are not known from the same locality (63 pairs);
— *pseudo-sympatric*: species pairs are found in the same region and do not overlap temporally, but are dated within 1 million years of one another (119 pairs);
— *contemporary*: species pairs overlap in their temporal range (a combination of sympatric and allopatric; 101 pairs); and
— *not sympatric*: species pair not known to overlap geographically within 1 million years temporally (815 pairs).

Padian and Horner suggested the ‘ghost of species recognition’ concept to test the species recognition hypothesis [3]; divergent clades with ‘exaggerated features’ need only to have been known to have coexisted. This hypothesis is untestable as presented because it does not require physical evidence of contemporary forms, and we cannot know if contemporary lineages were morphologically distinct without fossil evidence. Furthermore, all divergent lineages would, at some time, have been contemporary by definition. In an attempt to address the ‘ghost of species recognition’ concept [3], the contemporary category was created by merging the sympatric and allopatric categories. Categorizing species in this way ensures that fossil evidence is available for comparison of the morphology of contemporary species, and avoids speculating that morphological divergence had occurred at a chosen point.

To ensure that the relatively smaller datasets of the sympatry categories were not affecting comparison, we randomly sampled species pairs from the complete dataset to simulate populations of the same size as each sympatry category. This process was repeated 10 000 times for each category to provide a distribution of regression parameters, and the equivalent values for each sympatry category were compared with these distributions.

Finally, we assessed each character individually to test if sympatry was driving the divergence of individual traits. This was done by individually comparing the mean difference values of each character in the three main sympatry classes with the equivalent means in the remaining, non-sympatric species pairs.

## 3. Results

**Figure 2.**
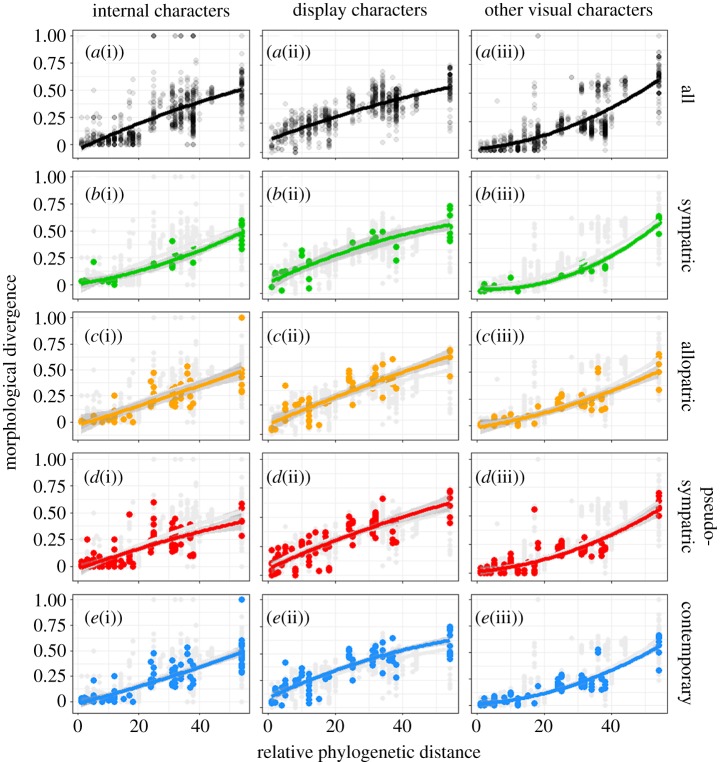
Pairwise comparison plots for sympatry categories (rows *a–e*) and character classes (columns i–iii). Species pairs that do not fall into sympatry categories are shown in light grey in rows *b* to *e* for reference. Second-order polynomial regressions fitted with confidence intervals are set at 95%.

All unique pairwise comparisons, excluding same-species comparisons 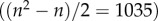, were plotted against relative phylogenetic distance (figure 2, row *a*). Trend lines in all cases are fitted with a second-order polynomial regression. This method captured the distribution of data while keeping the number of parameters low [38]. Morphological disparity increases with increasing relative phylogenetic distance, but the form of this relationship varies with the different classes of trait. The relatively low intercept values of internal (−0.028) and non-display characters (0.033), and shallower slope at low phylogenetic distances, suggest comparatively high levels of conservation of these characters between closely related species. By contrast, display characters show a higher intercept (0.100) across all species pairs (figure 2, cell *a*(ii)), and these characters are notably more divergent in closely related species pairs than are the internal or non-display characters.

Rows (*b*–*e*) of figure 2 show the divergence values for each character class when the species pairs are divided into the four sympatry categories. In the plots for the display characters, the allopatric and contemporary sympatry categories appear to depart slightly from the trend seen for all species pairs, but only at high relative phylogenetic distances. The trend lines for sympatric (*b*(i)) and pseudo-sympatric (*d*(i)) species pairs in the non-visual character class show a small deviation from the all-species trend at intermediate and high values of relative phylogenetic distance, respectively. This is not seen in the plots of this character class for other sympatry categories.

**Figure 3.**
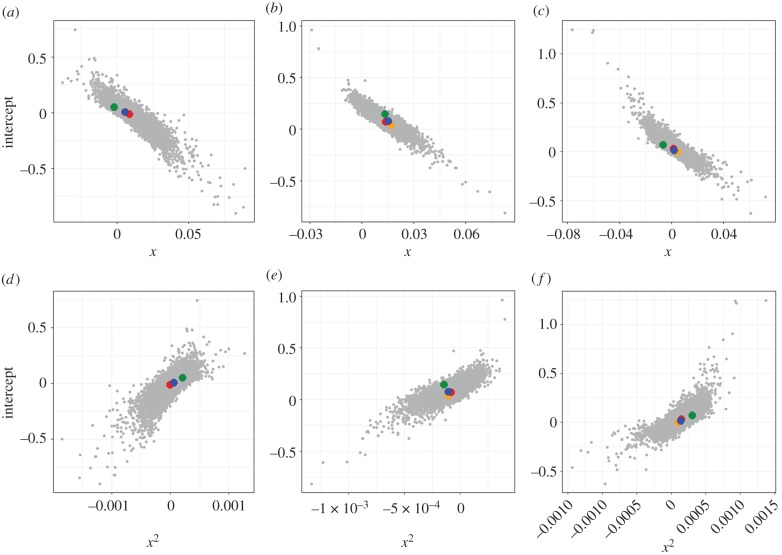
Distributions of second-order polynomial model output parameters for 10 000 randomly sampled *n* species pairs of internal (*a* and *d*), display (*b* and *e*), and other visual (*c* and *f*) character classes. Randomly sampled values are shown in grey. Values calculated for sympatry categories are overlaid in coloured points for each character class (green: sympatric; orange: allopatric; red: pseudo-sympatric; blue: contemporary).

The three second-order polynomial regression parameter values obtained for each of the observed character classes were plotted with the values obtained from random sampling for all species pairs (figure 3). All sympatry category parameters appear to fall well within the distribution of parameters from the randomly sampled datasets.

*Z*-scores were calculated for all the regression parameters, and all fell well within ±1.96 standard deviations (95% confidence interval) from the mean of the distribution of parameter values of the randomly sampled datasets (electronic supplementary material, table S2). This analysis, coupled with visual inspection of the plots in figure 2, suggests that sympatry does not have a significant effect on divergence of morphology in these groups.

Mean difference values for individual characters only show notably greater values in sympatric species pairs than other sympatry categories for two display characters (157 and 182; see the electronic supplementary material, figure S2 for details). These characters were found to be only present in 1 out of 38 and 3 out of 38 sympatric species pairs, respectively. It is likely that this is a result of the relatively small sample size of the sympatric category and does not reflect a genuine pattern of divergence among only these characters.

## 4. Discussion

We find no support for the hypothesis that sympatry correlates with higher ornament divergence in ceratopsian dinosaurs, nor for the wider species recognition explanation [3] for the evolution of horns, frills and other display traits in the Ceratopsia. Firstly, the divergence between ornaments of contemporary species is not significantly different from randomly sampled species, either individually or when considered as a suite of complimentary ornaments. This is true regardless of the level of sympatry, offering little support to the idea that the driver of ornament diversity was the need to differentiate between contemporary species. Secondly, although ornaments appear to show more rapid initial divergence than other structures, this pattern is not expected to be exclusive to species recognition-specific features; those involved in sexual selection or resource acquisition are known to undergo rapid morphological evolution and divergence [1,23,39].

A number of criticisms have been made of the species recognition hypothesis, chiefly that the drive to differentiate closely related species should evolve towards minimal cost [21]. Under the circumstances where two parties benefit from differentiating one another, low-cost signals are evolutionarily stable because neither party would benefit from either a dishonest or costly signal [40]. Although attempting to explain the divergence of ornamentation among ceratopsians, species recognition does not explain why all neoceratopsians evolved and maintained broadly similar structures in order to differentiate themselves from other species. The skulls of some neoceratopsians such as *Chasmosaurus* (figure 1*f*) have been shown to be such a large component of overall body mass that the centre of mass of the animal is shifted forwards to a degree that renders them obligately quadrupedal [41]. The amount of resources and energy required to grow and maintain such a structure is not consistent with a low-cost modification.

The few examples of species recognition-driven character divergence (as opposed to ecological character displacement) in extant species suggest low-cost modifications to pre-existing features that are common to both species in the non-overlapping portions of their ranges. Examples include facial markings in the rock nuthatches, *Sitta tephronata* and *Sitta neumayer* [15], plumage coloration in trogons [42] and the flycatcher *Ficedula* [18]*,* and dewlap coloration in the lizard genus *Anolis* [43]. No example is known of a large, costly structure evolving to serve primarily as a recognition signal in extant taxa [44]. Unique phenotypes may, of course, enable a member of a species to easily identify conspecifics, but for the examples known in extant taxa a different explanation of their origin exists [45]. An example is known of a so-called ‘social mimic’ in extant bird taxa, where two visually identical species overlap substantially in their ranges and are known to flock and forage together, but are nevertheless genetically distinct [46]. There is seemingly no impediment to their stable coexistence that warrants divergence of visual characters, as would be expected if species recognition were important.

An additional result of this study is in the comparison of curves for each character class across all species (figure 2, row *a*). The intercepts of the curves in both the *internal* (figure 2, *a*(i)) and *other visual* (figure 2, *a*(iii)) categories show a lower, near-zero, initial value than that seen in the *display* (figure 2, *a*(ii)) category. This difference suggests that traits associated with display in ceratopsians diverge at a quicker rate than do other features. This raises two possibilities: display features, implicated in visual communication, generally underwent rapid evolution during divergence of ceratopsian taxa, or that traits with a specific mechanical function (i.e. non-display characters; teeth, limbs, etc.) were under stronger stabilizing selection than display characters. The species recognition hypothesis does not appear to adequately account for this phenomenon because the pattern is a general trend observed across all included taxa, not simply those that are known or are implied to be sympatric. Visual signals are predicted to diverge rapidly under both sexual selection [1] and species recognition [3], but the effects of species recognition would only be seen in sympatric taxa where failure to distinguish conspecifics is costly.

The largest and most elaborate ceratopsian ornaments are found within the clade Ceratopsidea, including such well-known taxa as *Centrosaurus, Triceratops* and *Styracosaurus* (figure 1*c*,*g* and main image, respectively) [7]. The seemingly rapid and random divergence of skull ornamentation within this clade may point to one or several situations acting across it [47]. Firstly, interspecific selection acting in different directions would promote divergence between species. This may occur as a result of sympatric ecological niche partitioning, allopatric ecological selection, or as a result of the need for signalling diversity, as in species recognition [3,19]. Secondly, intraspecific mutually antagonistic coevolution where novelty is favoured creates conditions where arms races within species drive morphological evolution. An example of this is the evolution of weapons for use in intrasexual combat [48]. Thirdly, very flat selective landscapes create conditions where morphology is not constrained to evolve in a particular direction and so is free to evolve randomly from ancestral states. This last explanation seems unlikely given the high cost of ceratopsian ornaments, which were retained in almost all the species within the clade [7]. Niche partitioning and species recognition require some degree of interspecific interaction and, thus, sympatry. We have demonstrated here that sympatry has no significant effect on morphological disparity in ceratopsians. In the absence of any functional ecological role for ornamentation in ceratopsians [44], only intraspecific mutually antagonistic coevolution favouring novelty seems to apply to this clade, given the available evidence.

Sexual selection or social-sexual signalling has previously been proposed as a driver of ornament evolution and diversity in ceratopsians [1,2,21,44,49,50]. Most studies of sexually selected ornaments and weapons in extant taxa focus on sexually dimorphic examples [51,52], and there is no clear evidence of sexual dimorphism in Ceratopsia or any other dinosaur clade [10,53]. Except in extreme examples, large sample sizes are needed to distinguish sexes based on morphology alone [53,54]. Furthermore, mutual mate choice is also known to lead to the evolution of similar ornaments in both sexes in extant taxa [2,55]. If sexual selection is indeed the process behind the unique ornamentation of ceratopsians, it points to a set of conditions acting upon ceratopsians without obvious parallel in extant taxa. Nevertheless, sexually selected ornaments are expected to show characteristic patterns of growth, diversity and rapid evolution regardless of the taxa or characters in question [1]. Some support has been found for this in the positively allometric growth patterns of the ceratopsian *Protoceratops* [56], and the patterns of ornament divergence seen in Ceratopsia [7] are similar to those seen in the sexually selected horns of the scarab beetle genus *Onthophagus* [52]. Identifying these patterns is a challenge in fossil taxa, but an important step in the study of evolutionary palaeobiology, and evolutionary theory in general.

## Supplementary Material

Supplementary material

## Supplementary Material

Character matrix and phylogenetic distance data

## Data accessibility

Additional data can be found in the electronic supplementary material.
