# Supplementary material for: Patterns of divergence in the morphology of ceratopsian dinosaurs: sympatry is not a driver of ornament evolution
Source: Proc Biol Sci. 2018 Mar 21;285(1875):20180312. doi: 10.1098/rspb.2018.0312 (PMC5897650; doi:10.1098/rspb.2018.0312)
Supplement: Supplementary material [file rspb20180312supp1.pdf]

Patterns of divergence in the morphology of ceratopsian dinosaurs, with an analysis of sympatry as a driver of divergence.

A Knapp, RJ Knell, AA Farke, MA Loewen and DWE Hone

Supplementary material

Contents

Figure S1: Time-scaled phylogeny of Ceratopsia

Table S1: Temporal dates and location information for all ceratopsians used in study

Table S2: Z-scores and associated *p* values for randomisation test

Figure S2: Mean values for individual characters

List of morphological characters used in difference calculation

Supplementary material references

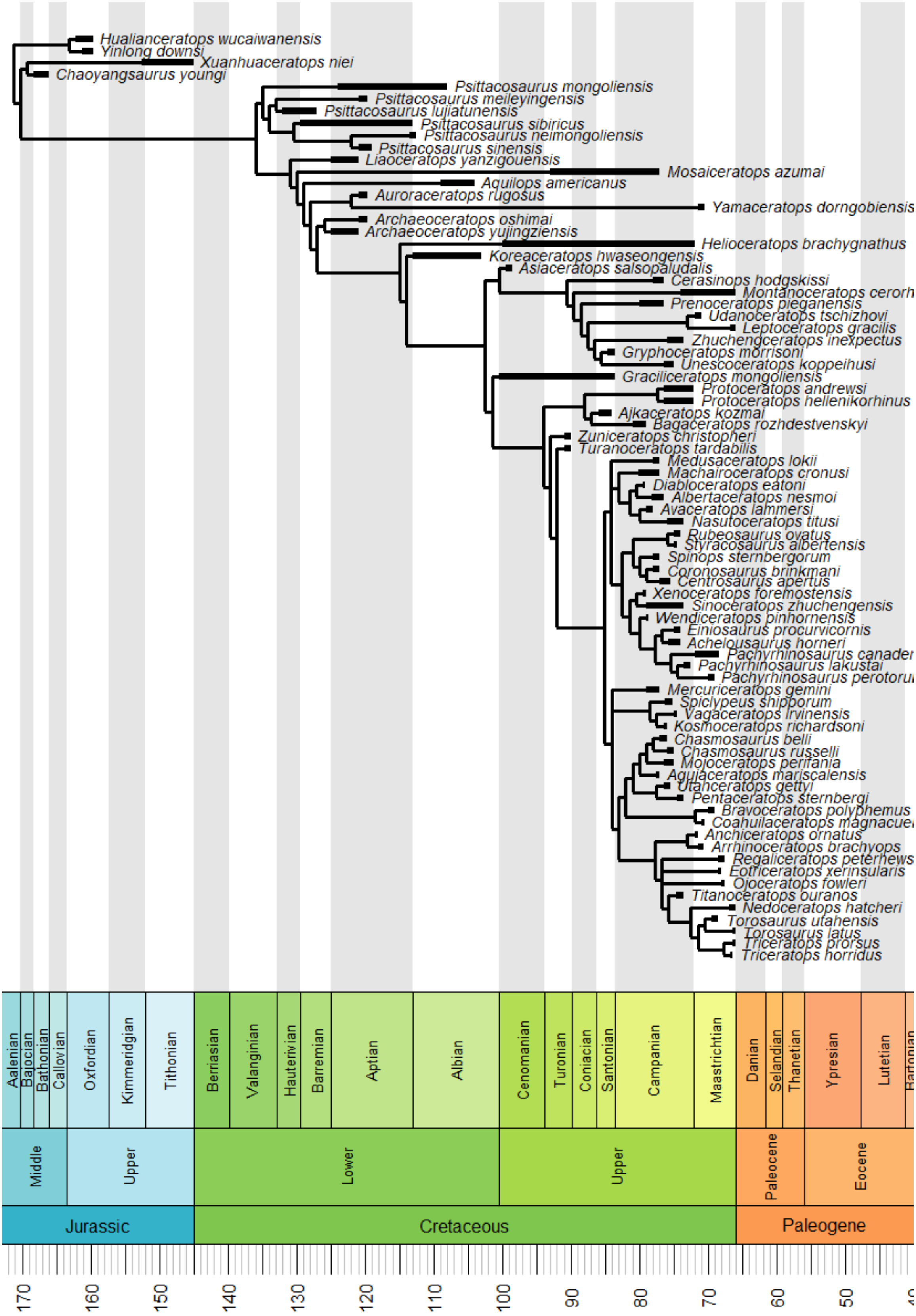

Figure S1: Time-scaled phylogeny for all known ceratopsian species, used to calculate relative phylogenetic distances. Estimated species ranges are indicated by thick bars at branch tips. Created using strap package for R (Bell and Lloyd, 2014).

Table S1: Temporal calibrations and geographical locations of ceratopsian species. Taxa that are included in the morphological character state analysis are indicated in bold type. Region abbreviations: Asia: A; North America: NA; Europe: E.

**Taxon**	Region	Lower Bound	Upper Bound	Source
Yinlong downsi	A	161.2	155.7	Xu et al., 2006
Chaoyangsaurus youngi	A	168.3	166.1	Zhao et al., 1999
Hualianceratops wucaiwnensis	A	162.2	159.7	Han et al., 2015
Xuanhuaceratops niei	A	152.6	145	Zhao et al., 2006
Psittacosaurus lujiatunensis	A	132	127	Swisher et al., 1999; Changfu et al., 2006
Psittacosaurus mongoliensis	A	124	108	Sereno, 2010
Psittacosaurus neimongoliensis	A	113.5	112.5	Sereno, 2010
Psittacosaurus meileyingensis	A	121	119.6	Sereno, 2010
Psittacosaurus sibiricus	A	129.4	113	Sereno, 2010
Psittacosaurus sinensis	A	121	119	Gao and Cheng, 1999; Sereno, 2010
Mosaiceratops azumai	A	93	77	Zheng, Jin and Xu, 2015
Liaoceratops yangzigouensis	A	125	121	Xu et al., 2002
Yamaceratops dorngobiensis	A	71.5	70.5	Shuvalov, 2000
Auroraceratops rugosus	A	121	119.6	You et al., 2005
Archaeoceratops yujingziensis	A	125	121	You, Tanoue and Dodson, 2010
Archaeoceratops oshimai	A	121	119.6	You et al., 2003
Aquilops americanus	NA	109	104	Farke et al., 2014
Unescoceratops koppeihusi	NA	76.5	75.8	Ryan et al., 2012
Grypoceratops morrisoni	NA	84.6	83.6	Ryan et al., 2012
Zhuchengceratops inexpectus	A	76	73.5	Xu et al., 2010
Helioceratops brachygnathus	A	100	72	Liyong et al., 2009
Koreaceratops hwaseongensis	A	113	103	Lee et al., 2010
Asiaceratops salsopaludalis	A	99.5	98.5	Ryan et al., 2012
Cerasinops hodgkissi	NA	78	76.5	Ryan et al., 2012
Montanaceratops cerorhynchus	NA	74	66	Chinnery and Weishampel, 1998
Prenoceratops pieganensis	NA	80	76.5	Ryan et al., 2012
Leptoceratops gracilis	NA	66.8	66	Ott, 2007
Udanoceratops tschizhovi	A	72	71	Dodson, 1996
Graciliceratops mongoliensis	A	100.5	83.6	Maryańska and Osmólska, 1975; Hicks et al, 1999
Protoceratops andrewsi	A	76.38	72.05	Shuvalov, 2000; Dashzeveg et al., 2005
Protoceratops hellenikorhinus	A	76.38	72.05	Shuvalov, 2000; Lambert, 2001; Dashzeveg et al., 2005
Ajkaceratops kozmai	E	86	84	Osi et al., 2010
Bagaceratops rozhdestvenskyi	A	81	79	Maryańska and Osmólska, 1975
Turanoceratops tardabilis	A	91	90	Sues and Averianov, 2009
Zuniceratops christopheri	A	91	90	Wolfe et al., 2010

Diabloceratops eatoni	NA	79.5	79.1	Kirkland and Deblieux, 2010; Roberts et al., 2013
Machairoceratops cronusi	NA	80.1	77	Lund et al., 2016
Albertaceratops nesmoi	NA	78.2	76.4	Ryan, 2002
Medusaceratops lokii	NA	78	77	Ryan, Russell and Hartman, 2010
Xenoceratops formostensis	NA	79.5	79	Ryan, Evans and Shepherd, 2012
Wendiceratops pinhornensis	NA	79	78.7	Evans and Ryan, 2015
Sinoceratops zhuchengensis	A	79	73.5	Xu et al., 2010
Nasutoceratops titusi	NA	76	75.5	Lund, 2010
Avaceratops lammersi	NA	79	78	Dodson, 1986
Spinops sternbergorum	NA	78	77	Farke et al., 2011
Coronosaurus brinkmani	NA	78	77	Ryan and Russell, 2005
Centrosaurus apertus	NA	77	75.5	Arbour, Burns and Sissons, 2009
Styracosaurus albertensis	NA	75	74.5	Sampson and Loewen, 2010
Rubeosaurus ovatus	NA	75	74	McDonald and Horner, 2010
Einiosaurus procurvicornis	NA	75	74	Sampson, 1995
Achelousaurus horneri	NA	75.8	74	Sampson, 1995
Pachyrhinosaurus canadensis	NA	72	68.3	Ryan et al., 2010
Pachyrhinosaurus lakustai	NA	73.5	72.5	Currie, Langston and Tanke, 2008
Pachyrhinosaurus perotorum	NA	70	69	Fiorillo and Tykoski, 2011
Kosmoceratops richardsoni	NA	76.5	76	Sampson et al., 2010
Spiclypeus shipporum	NA	76.2	75.2	Mallon et al., 2016
Vagaceratops irvinensis	NA	75	74.5	Sampson and Loewen, 2010
Agujaceratops mariscalensis	NA	77.5	77	Sampson et al., 2010
Mercuriceratops gemini	NA	79	77	Ryan et al., 2014
Mojoceratops perifania	NA	76.5	75	Longrich, 2010
Chasmosaurus belli	NA	77	76	Sampson et al., 2010
Chasmosaurus russelli	NA	76	75	Sampson et al., 2010; Longrich, 2010
Utahceratops gettyi	NA	76.5	75.5	Sampson et al., 2010
Pentaceratops sternbergi	NA	74.5	73.5	Sampson et al., 2010
Coahuilaceratops magnacuerna	NA	71	70.5	Sampson et al., 2010
Anchiceratops ornatus	NA	72	71.5	Sampson et al., 2010
Bravoceratops polyphemus	NA	70	69	Wick and Lehman, 2013
Regaliceratops peterhewsi	NA	68.5	67.5	Brown and Henderson, 2015
Arrhinoceratops brachyops	NA	71.5	70.6	Sampson and Loewen, 2010
Ojoceratops fowleri	NA	68	67.5	Sampson et al., 2010
Titanoceratops ouranos	NA	74.7	73.6	Longrich, 2011
Torosaurus latus	NA	66.8	66	Farke, 2007
Torosaurus utahensis	NA	69.5	68.5	Sullivan, Boere and Lucas, 2005
Eotriceratops xerinsularis	NA	68.5	68	Sampson et al., 2010

Nedoceratops hatcheri	NA	67	66	Farke, 2011
Triceratops horridus	NA	66.8	66.4	Sampson et al., 2010 ; Scanella et al., 2014
Triceratops prorsus	NA	66.4	66	Renne et al., 2013 ; Scanella et al., 2014

Table S2: z-scores of second-order polynomial regression parameters carried out on observed species pairs vs. 10000 randomly-sampled species pairs of number *n* for each sympatry category. Values are listed for each sympatry category, as outlined in the introduction. All parameters fall within ±1.96 standard deviations, representing a 95% confidence interval.

	**Internal**						
		intercept		x		x^2	
	n	*z*	*p*	*z*	*p*	*z*	*p*
Sympatric	38	0.65	0.516	-0.99	0.322	0.80	0.424
Allopatric	63	-0.14	0.889	-0.16	0.873	0.08	0.936
Pseudosympatric	119	-1.41	0.159	-0.51	0.610	0.00	1.00
Contemporary	101	0.39	0.697	-0.73	0.465	0.53	0.596

	**Display**						
		intercept		x		x^2	
	n	*z*	*p*	*z*	*p*	*z*	*p*
Sympatric	38	-0.07	0.944	0.24	0.810	-0.06	0.952
Allopatric	63	-0.30	0.764	0.73	0.465	-0.02	0.984
Pseudosympatric	119	-1.36	0.174	0.88	0.379	-0.21	0.834
Contemporary	101	-0.45	0.653	1.16	0.246	-0.52	0.603

	**Other visual**						
		intercept		x		x^2	
	n	*z*	*p*	*z*	*p*	*z*	*p*
Sympatric	38	-0.01	0.992	-0.79	0.430	0.70	0.484
Allopatric	63	-0.96	0.337	0.98	0.327	-1.24	0.215
Pseudosympatric	119	-0.76	0.447	0.20	0.841	-0.49	0.624
Contemporary	101	-0.36	0.719	0.39	0.697	-0.58	0.562

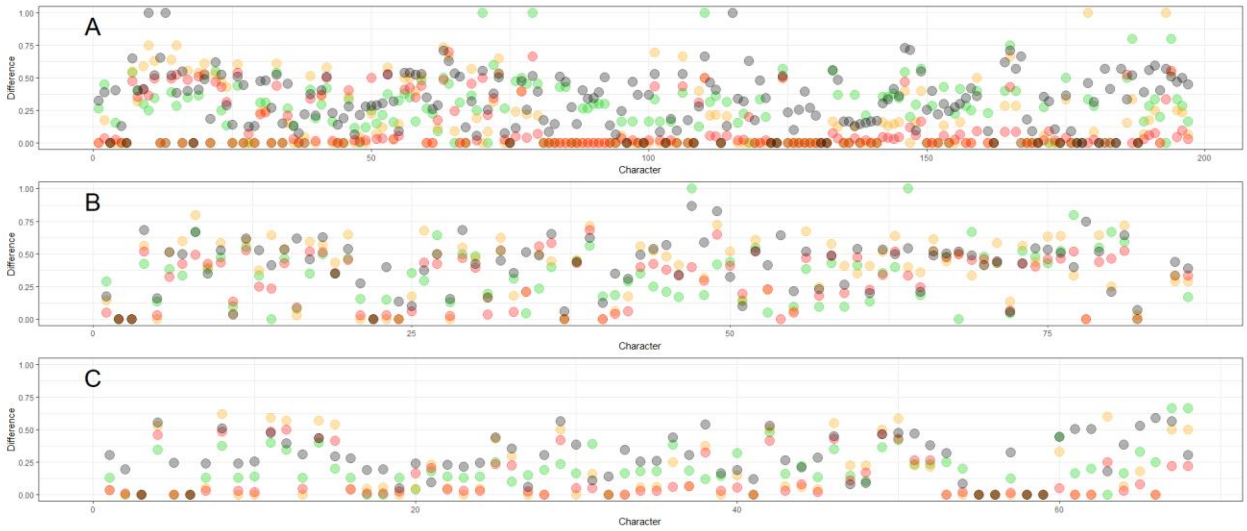

Figure S2*:* Means difference values of each individual character for each main sympatry category for internal (A), display (B), and other visual (C) character classes (n.b. here, characters are numbered according to order rather than their corresponding numbers from the character list). Means are shown for sympatric (green), allopatric (orange), pseudosympatric (red) and all other (black) species pairs. Characters range from 0 (all identical) to 1 (all different). In only 6 out of a total of 86 display characters (B) do sympatric species pairs show the highest average, and only two of these have a mean value of 1, indicating consistent difference. These two characters are represented by only one and three sympatric species pairs, and so are likely to be anomalous due to small sample size. The lack of any clear pattern in the display characters plot (B) does not suggest that individual characters are used as distinguishing traits in sympatric species. Internal characters (A) also show universal differences (mean value = 1.00) in three instances. As with display characters, these are associated with small numbers of species pairs sharing known states in each character (Characters 252, 262, and 296, representing two, two and one species pairs for each character respectively).

List of morphological characters used in difference calculation

General Skull

1. Skull, size relative to the body:
(0) – small, shorter than the length of the upper hindlimb bones
(1) – sub-equal to the length of the femora
(2) – large, at least 150% of the length of the upper limb bones
(Modified from Makovicky and Norell, 2006:1)

2. Skull, shape of facial skull in dorsal view:
(0) – elongated and ovoid
(1) – triangular, wide over the jugals
(Makovicky and Norell, 2006:2)

3. Skull, relative height of snout (premaxilla) to orbital region:
(0) – low
(1) – deep
(Modified from Makovicky and Norell, 2006:10)

4. Skull, naris length compared to basal skull length:
(0) – less than 10% of skull length
(1) – more than 10% but less than 15% of skull length

(2) – more than 18% of skull length
(Modified from Makovicky and Norell, 2006:19)

5. **Skull, preorbital skull length compared to overall skull length (disregarding spikes):**
(0) – more than 41%
(1) – 35 to 40%
(2) – 30 to 34%
(3) – less than 29%
(Modified from Makovicky and Norell, 2006:4)

6. **Palatomaxillary foramen:**
(0) – absent
(1) – present
(Hatcher et al., 1907)

7. **Position of anterior end of choana on palate:**
(0) – anterior to maxillary tooth row
(1) – or even with maxillary tooth row
(Makovicky & Norell, 2006:20)

8. **Skull, suborbital fenestra or pterygopalatine foramen size:**
(0) – large
(1) – diminutive
(Modified from Makovicky and Norell, 2006:45

Rostral

9. **Rostral, presence of the rostral forming beak:**
(0) – absent
(1) – present
(Modified from Makovicky and Norell, 2006:6)

10. **Rostral, shape of body and extent of dorsal and ventral processes:**
(0) – triangular in lateral view, lacks discrete dorsal and ventral processes
(1) – elongated dorsal and ventral processes extending from the rostral body with prominent concavity on the posterior margin invaginated at least 33% or anteroposterior length of the element
(2) – short, broad dorsal and ventral processes
(Dodson et al., 2004:1; Sampson et al., 2010:1; Farke et al., 2011:1)

11. **Rostral, presence of ventral (buccal) process:**
(0) – absent
(1) – present
(Modified from Makovicky and Norell, 2006:7)

12. **Rostral, contact with nasal:**
(0) – present
(1) – absent
(New Character)

13. **Rostral, shape in occlusal view:**
(0) – broad and rounded anteriorly, “U” shaped
(1) – acute, “V”-shaped
(New Character)

Premaxilla

14. **Premaxilla, position of ventral border of endonaris compared to infratemporal fenestra:**
(0) – significantly above ventral rim of infratemporal fenestra
(1) – about the level of ventral rim of infratemporal fenestra
(2) – significantly below ventral rim of infratemporal fenestra
(Modified from Makovicky & Norell, 2006:16) Ventral border of ectonaris

15. **Premaxilla, position of ventral border of nares compared to orbit:**

(0) – is positioned well above the ventral extent of the orbit
(1) – extends to just above the ventral extent of the orbit but not below
(2) – extends below the orbit
(Modified from Makovicky & Norell, 2006:15)

16. Premaxilla, position of ventral border of nares compared to alveolar tooth margin:
(0) – extends below the orbit but not to the alveolar margin
(1) – extends to the alveolar margin
(Modified from Makovicky & Norell, 2006:15)

17. Premaxilla, presence of ectonaris:
(0) – absent
(1) – present as slight outline on lateral surface of premaxilla
(2) – present as a discrete invagination
(Modified from Makovicky and Norell, 2006:17; Sampson et al., 2010:4)

18. Premaxilla, ectonaris, shape:
(0) – anteriorly elongate
(1) – tall, hemicircular
(Sampson et al., 2010:4; Farke et al., 2011:2)

19. Premaxilla, osteonarial complex, size:
(0) – small, occupies 50% or less height of snout
(1) – large, occupies entire height of snout
(Modified after Gregory & Mook, 1925; Brown & Schlaikjer, 1940a)

20. Premaxilla, presence of premaxillary septum:
(0) – absent
(1) – present
(Forster, 1990)

21. Premaxillary septum, nasal contribution:
(0) – septum formed by premaxilla only
(1) – septum formed by premaxilla and nasal
(Sampson et al., 2010:5; Farke et al., 2011:3)

22. Premaxilla, narial strut:
(0) – absent
(1) – present, nascent
(2) – present, well-developed
(Farke et al., 2011:4)

23. Premaxilla, narial strut orientation:
(0) – anteriorly inclined
(1) – posteriorly inclined
(Dodson et al., 2004:6; Sampson et al., 2010:7)

24. Premaxilla, septal flange:
(0) – absent
(1) – fin of bone posterior to narial strut present
(Modified from Forster et al., 1993:1)

25. Premaxilla, septal flange length:
(0) – spans entire posterior margin of narial strut
(1) – restricted to ventral portion of narial strut
(Modified from Forster et al., 1993: 1)

26. Premaxilla, septal fossa:
(0) – absent
(1) – present
(Farke et al., 2011:5)

27. Premaxilla, septal recess:
(0) – absent
(1) – present

(Modified from Dodson et al., 2004:9; Farke et al., 2011:7)

28. **Premaxilla, accessory strut in septal fossa and septal recess:**
(0) – absent
(1) – present
(Sampson et al., 2010:12)

29. **Premaxilla, endonarial process:**
(0) – absent
(1) – (triangular process) present
(Forster, 1990:21; Sampson et al., 2010:13; Farke et al., 2011:6)

30. **Premaxilla, endonarial process shape in lateral view:**
(0) – square with straight margins
(1) – triangular with concave margins
(Sampson et al., 2010:14)

31. **Premaxilla, endonarial process recess:**
(0) – absent
(1) – present
(Forster, 1990: XX; Sampson et al., 2010:15)

32. **Premaxilla, ectonarial recess**
(0) – absent
(1) – present
(Sampson et al., 2010:16)

33. **Premaxilla, endonarial platform:**
(0) – present
(1) – absent
(New Character)

34. **Premaxilla, ventral palatal region shape:**
(0) – flat in ventral view
(1) – vaulted dorsally
(Modified from Makovicky and Norell, 2006:9)

35. **Premaxilla, ventral extent compared to level of alveolar margin:**
(0) – above or at the level of alveolar margin of maxilla
(1) – expanded ventrally to extend well below alveolar margin of maxilla
(Modified after Makovicky & Norell, 2006:12; Farke et al., 2011:9)

36. **Premaxilla, presence of ventral angle:**
(0) – absent
(1) – present
(Forster, 1990; Sampson et al., 2010:17; Farke et al., 2011:8)

37. **Premaxilla, composition of expanded ventral angle:**
(0) – premaxilla and maxilla
(1) – premaxilla
(Modified after Makovicky & Norell, 2006:12; Farke et al., 2011:10)

38. **Premaxilla, form of distal end of posterioventral process:**
(0) – broad and uniform in width
(1) – broad and expanded, paddle shaped
(2) – thin
(Modified from Forster et al., 1993:7; Farke et al., 2011:12)

39. **Premaxilla, form of thin distal end of posterioventral process:**
(0) – lancelate
(1) – forked
(Modified from Forster et al., 1993:7; Sampson et al., 2010:18; Farke et al., 2011; 11)

40. **Premaxilla, nasal dorsal contact in dorsal view:**
(0) – premaxillae inserts between nasals
(1) – nasals insert between premaxillae
(Sampson et al., 2010:20, Farke et al, 2011:13)

41. **Premaxilla-nasal dorsal contact, length in dorsal view:**
(0) – short
(1) – premaxilla and nasal contact for at least 30% of the length of the nasal
(New Character)

42. **Premaxilla, premaxilla-nasal contact in dorsal view:**
(0) – premaxilla tapers posteriorly
(1) – premaxilla broadens posteriorly
(New Character)

43. **Premaxilla, contribution of premaxilla to posterior margin of external naris**
(0) – less than 40%
(1) – more than 45%
(New Character)

44. **Premaxilla, premaxilla-prefrontal contact:**
(0) – absent
(1) – present
(Modified from Makovicky and Norell, 2006:11)

Accessory Antorbital Fenestra

45. **Accessory antorbital fenestra, presence between naris and antorbital fenestra:**
(0) – absent
(1) – present
(Forster 1990; 15; Sampson et al., 2010:21; Farke et al., 2011:14; Farke et al., 2014:143)

46. **Accessory antorbital fenestra size.**
(0) – pronounced, penetration of nasal cavity visible in lateral view
(1) – slight penetration, nasal cavity not visible in lateral view
(Sampson et al., 2010:22; Farke et al., 2011:15)

47. **Accessory antorbital fenestra, nasal contribution to dorsal margin:**
(0) – nasal makes up less than 50%
(1) – nasal makes up more than 60%
(New Character)

Antorbital Fenestra

48. **Antorbital fenestra:**
(0) – present
(1) – nascent or vestigial
(2) – absent
(New Character)

49. **Antorbital fenestra exposure in lateral view:**
(0) – well exposed in lateral view
(1) – fenestra is tucked behind flange of the jugal
(New Character)

50. **External antorbital fenestra/fossa, size:**
(0) – large, 20% or more length of body of maxilla
(1) – greatly reduced, less than 10% length of body of maxilla
(Modified from Granger & Gregory, 1923; Sampson et al., 2010:23; Farke et al., 2011:16; Makovicky and Norell, 2006:23)

Maxilla

51. **Maxilla, shape of oral margin in ventral view:**
(0) – sinuous, laterally concave
(1) – relatively straight, oral margins converge anteriorly
(Modified Makovicky & Norell, 2006:13)

52. **Maxilla, ventral displacement of tooth row relative to ventral surface of premaxilla:**

(0) – absent
(1) – present
(Sampson et al., 2010:24; Farke et al., 2011:17)

53. Maxilla, tooth row diastema:
(0) – absent
(1) – present
(Sampson et al., 2010:25)

54. Maxilla, maxillary cavity opening on the medial surface of the ascending process and penetrating into the interior of the bone:
(0) – absent
(1) – present
(Sampson et al., 2010:26; Farke et al., 2011:18)

55. Maxilla, internal maxillary fossa on medial surface dorsal to the palatal process:
(0) – absent
(1) – present
(New Character)

56. Maxilla, length of posterior (alveolar) ramus compared to toothrow length:
(0) – short, 25% or less
(1) – long, 30% or more
(Sereno, 1999)

57. Maxilla, ectotopterygoid/pterygoid complex exposure on posterior ramus:
(0) – covers entire dorsal surface and laps onto lateral surface
(1) – reduced to the posterior-most portion
(Farke et al., 2011:19)

58. Maxilla, relation to vomers:
(0) – separated by vomers
(1) – contact each other in palatal view; tip of vomer obscured from view
(Modified from Makovicky and Norell, 2006:22)

59. Maxilla, eminence or tubercle on the rim of the buccal emargination of the maxilla near the junction with the jugal:
(0) – absent
(1) – present
(Modified from Makovicky and Norell, 2006:24)

Nasal

60. Nasal, anterior end relative to anterior end of external naris:
(0) – posterior to
(1) – anterior to
(Modified from Makovicky and Norell, 2006:14)

61. Nasal, dorsal surface nasal-nasal contact transverse section:
(0) – concave
(1) – flat
(2) – peaked or convex
(Modified from Farke et al., 2014:137)

62. Nasal, ornamentation in adult:
(0) – absent
(1) – present
(Forster, 1990; Sampson et al., 2010:27)

63. Nasal, ornamentation type in adult:
(0) – low prominence
(1) – distinct horncore
(2) – pachyostotic boss
(Modified from Forster, 1990: 26,27,28,126; Sampson et al., 2010:28; Farke et al., 2011:20)

64. Nasal, ornamentation position, measured perpendicular to the toothrow:
(0) – centered posterior to anterior end of toothrow
(1) – centered anterior to anterior end of of toothrow
(Modified after Forster, 1990; Sampson et al., 2010:29)

65. Nasal ornamamentation base, cross sectional shape:
(0) – sub-circular to ovoid
(1) – mediolaterally compressed, width 66% anteroposterior length of base
(2) – elongate width less than 33% anteroposterior length of base
(3) – covers entire nasal
(New Character)

66. Nasal ornamentation, height compared to the orbit:
(0) – short, less than or equal to the height of the orbit
(1) – 1.5 to 2 times taller than the orbit
(2) – elongated, more than 3 times taller than the orbit
(New Character)

67. Nasal, epinasal ossification:
(0) – absent
(1) – present
(Lehman, 1990; Forster, 1990; Farke et al., 2011:21)

68. Nasal, dorsoventral thickness at apex of ectonaris:
(0) – thin, uniform thickness from medial to lateral
(1) – thick, ventral surface of nasal relatively flat
(New Character)

69. Nasal, vaulting of internal surface:
(0) – flat
(1) – absent, convex ventrally
(2) – present, ventral surface concave
(New Character)

70. Nasal, narial spine:
(0) – absent
(1) – present as a distinct inflection in the posterior or posteriomedial surface of the naris
(Modified from Lehman, 1990; Forster, 1990:22; Sampson et al., 2010:30, Farke et al., 2011:22)

71. Nasal, contribution to narial spine:
(0) – forms less than 60 % of narial spine
(1) – forms greater than 70% of narial spine
(New Character)

72. Nasal, narial spine orientation:
(0) – nascent process medially directed
(1) – process anteriorly directed
(New Character)

Orbit

73. Orbital region, facial skeleton, dorsoventral depth:
(0) – deep, maxilla dorsally expanded, posterior ramus of maxilla entirely visible
(1) – shallow, maxilla dorsoventrally low, posterior ramus of maxilla partially covered by jugal
(Sampson et al., 2010:31)

74. Orbit, orientation:
(0) – directed anterolaterally
(1) – directed laterally
(Sampson et al., 2010:32)

75. Orbit diameter, relative to basal skull length:
(0) – more than 25%
(1) – less than 20%

(Makovicky & Norell, 2006:3; Sampson et al., 2010:33)

76. Orbit, contribution of lacrimal to orbital margin:
(0) – forms 50% or more of the margin
(1) – forms 40% or less of the margin
(Brown and Schlaikjer, 1940a; Sampson et al., 2010:34)

Palpebal

77. Palpebral, shape and attachment:
(0) – rod-like, articulates with prefrontal only at its base, ligamentous attachment
(1) – blocky, fully sutured to and fused into prefrontal and frontal around dorsal orbital margin
(Granger & Gregory, 1923; modified from Forster 1990:31; Makovicky and Norell, 2006:25; Sampson et al., 2010:43; Farke et al., 2011:32)

78. Palpebral, antorbital buttress:
(0) – absent
(1) – present
(Ostrom and Wellnhofer, 1986; Sampson, 1995:7: Sampson et al., 2010:44; Farke et al., 2011:33)

79. Palpebral, extent of antorbital buttress:
(0) – restricted to anterodorsal orbital margin
(1) – present along entire anterior orbit al margin
(Ostrom and Wellnhofer, 1986; Sampson, 1995a: Sampson et al., 2010:45; Farke et al., 2011:34)

Postorbital

80. Postorbital, overhang projecting into supratemporal fenestra:
(0) – absent
(1) – present
(Makovicky & Norell, 2006:33)

81. Postorbital, texture of lateral surface on orbital rim:
(0) – smooth
(1) – strongly sculptured
(Modified from Farke et al., 2014:134)

82. Postorbital, supraorbital ornamentation in adult:
(0) – absent
(1) – low ornamentation
(2) – horncore
(Modified from Forster et al., 1993:5; Makovicky and Norell, 2006:31; Sampson et al., 2010:3; Farke et al., 2011:25 and 28)

83. Postorbital, form of low supraorbital ornamentation in adult:
(0) – reduced to low rugosity
(1) – pachyostotic boss
(Modified from Forster et al., 1993:5; Sampson et al., 2010:3; Farke et al., 2011:25 and 28)

84. Postorbital, type of subadult supraorbital ornamentation:
(0) – pointed apex, horncore at least as tall as long anteroposteriorly
(1) – rounded apex, horncore longer anteroposteriorly than high
(Modified from Sampson, 1995a:10; Farke et al., 2011:24)

85. Postorbital ornamentation, size relative to the nasal ornamentation:
(0) – larger than nasal ornamentation
(1) – smaller than nasal ornamentation
(New Character)

86. Postorbital, position of supraorbital ornamentation:
(0) – centered anterodorsal or dorsal to orbit, narrow base with posterior margin of supraorbital horn extending to or only slightly behind posterior margin of orbit
(1) – centered posteriodorsal to orbit, broad base with posterior margin of supraorbital horn extending well behind posterior orbit

(Modified from Lehman, 1996; Sampson et al., 2010:37; Farke et al., 2011:26)

87. **Postorbital, orientation of supraorbital horncore base:**
(0) – dorsally directed
(1) – dorsolaterally directed
(Sampson et al., 2010:38; Farke et al., 2011:27)

88. **Postorbital, curvature of supraorbital horncore of adults in lateral view:**
(0) – posteriorly curved
(1) – anteriorly curved
(2) – straight or too small to have curvature
(Modified from Forster et al., 1993:2; Sampson et al., 2010:40; Farke et al., 2011:29)

89. **Postorbital, curvature of supraorbital horncore of adults in anterior view:**
(0) – medially curved
(1) – laterally curved
(2) – straight or too small to have curvature
(Sampson et al., 2010:41, Farke et al., 2011:30)

90. **Postorbital, cornual sinuses in base of supraorbital ornamentation:**
(0) – absent
(1) – present
(Farke, 2006)

91. **Postorbital, extent of cornual sinuses in base of supraorbital ornamentation:**
(0) – sinus space invades frontal and parietal only
(1) – sinus space enters postorbital
(Modified from Forster 1990: 123; Farke, 2006; Sampson et al., 2010:36; Farke et al., 2011:23)

92. **Postorbital, contribution to laterotemporal fenestra:**
(0) – postorbital participation in margin
(1) – postorbital excluded from margin
(Makovicky & Norell, 2006:34)

93. **Postorbital, separation from laterotemporal fenestra:**
(0) – postorbital excluded from margin slightly by narrow strip of jugal
(1) – jugal–squamosal contact very wide and postorbital situated far from fenestra
(Makovicky & Norell, 2006:34; Sampson et al., 2010:42)

Jugal

94. **Jugal, shape of jugal flange:**
(0) – free jugal flange absent
(1) – broad based, narrows distally (Delta-shaped)
(2) – narrowed base, anterior and posterior margins subparallel (narrow-waisted)
(New Character)

95. **Jugal, thickening of jugal flange along its anterior edge:**
(0) – present
(1) – absent, plate-like flange
(New Character)

96. **Jugal, size and orientation of jugal flange:**
(0) – projects strongly posteriorly, distal end does not extend below maxillary tooth row
(1) – projects more ventrally, distal end extends below maxillary tooth row
(Makovicky 2001:22; Sampson et al., 2010:46; Farke et al., 2011:35)

97. **Jugal, suborbital ramus depth:**
(0) – not as deep as subtemporal ramus
(1) – as deep or deeper than subtemporal ramus
(Modified from Makovicky and Norell, 2006:28)

98. **Jugal infratemporal process:**
(0) – absent
(1) – present

(Modified from Brown and Schlaikjer, 1940a; modified from Forster, 1990:62; Sampson et al., 2010:47; Farke et al., 2011:36)

99. **Jugal infratemporal process:**
(0) – long
(1) – short
(Modified from Brown and Schlaikjer, 1940a; modified from Forster, 1990:62; Sampson et al., 2010:47; Farke et al., 2011:36)

100. **Jugal–lacrimal contact:**
(0) – very limited
(1) – extensive
(Makovicky & Norell, 2006:26; Sampson et al., 2010:48)

101. **Jugal horn orientation:**
(0) – jugal ornamentation lacking
(1) – laterally directed
(2) – ventrolaterally directed
(Makovicky & Norell, 2006:27)

Epijugal

102. **Epijugal**:
(0) – absent
(1) – present
(Makovicky and Norell, 2006:29)

103. **Epijugal, position of epijugal on jugal:**
(0) – covers most of the posterodistal jugal
(1) – restricted to just the distal most tip of the jugal
(Modified from Makovicky and Norell, 2006:30)

104. **Epijugal cross-sectional shape:**
(0) – large blade like triangle with obtuse angle oriented towards quadratojugal
(1) – roughly equilateral in shape
(Sampson et al., 2010:49; Farke et al., 2011:37)

105. **Epijugal length of horn:**
(0) – as long or longer than wide
(1) – substantially shorter than wide
(Modified from Sampson et al., 2010:50)

106. **Epijugal, extent of horn length:**
(0) – as long as wide
(1) – at least 1.5 times longer than wide
(New Character)

Quadratojugal

107. **Quadratojugal shape:**
(0) – mediolaterally compressed
(1) – transversely expanded medial to jugal
(2) – a squat mediolaterally flattened pedestal
(Modified from Makovicky & Norell, 2006:40)

108. **Quadratojugal, shape of contact with jugal:**
(0) – undivided
(1) – bifid around posterior end of jugal
(Modified from Farke et al., 2006:136)

109. **Quadratojugal, contact with squamosal:**
(0) – absent
(1) – present
(Brown and Schlaikjer, 1940a; Sampson et al., 2010:51)

110. Laterotemporal fenestra, position:
(0) – extends dorsal to ventral margin of orbit
(1) – positioned entirely below ventral limit of orbit
(Brown and Schlaikjer, 1940a; Forster, 1990)

111. Laterotemporal fenestra, size:
(0) – huge, subequal in size to orbit with long axis at least 50% basal skull length
(1) – greatly reduced, much smaller than orbit, no more than 30% skull length,
(Modified after Brown and Schlaikjer, 1940a; Forster, 1990; Makovicky and Norell, 2006:35)

Prefrontal and Frontal

112. Prefrontal, both prefrontals contact at the midline:
(0) – absent
(1) – present
(Lambe, 1915; Forster, 1990:30; Sampson, 1995a; Farke et al., 2011:31)

113. Frontal, contribution to orbital margin:
(0) – present
(1) – absent
(Brown & Schlaikjer, 1940a; Sampson et al., 2010:53)

114. Frontal, contribution to supratemporal fenestra:
(0) – present
(1) – absent
(Lambe, 1915; Sampson et al., 2010:54)

115. Frontal, supracranial cavity complex:
(0) – absent
(1) – present, shallow depression
(2) – present, rimmed
(Modified from Forster 1990:3; modified from Makovicky and Norell, 2006:51; modified from Sampson et al., 2010:55; modified from Farke et al., 2011:38)

116. Frontal fontanelle, shape:
(0) – present, transversely narrow, slit-like
(1) – present, key-hole shaped, circular or elongate oval
(Modified from Forster 1990:49,50; Forster, 1996a:3; Sampson et al., 2010:56; Farke et al., 2011:39)

Squamosal

117. Squamosal-quadrate contact:
(0) – socket-like cotylus on ventrolateral squamosal for ball-like quadrate head
(1) – elongate groove on medial surface of squamosal to receive lamina of quadrate
(Hatcher et al., 1907; Forster, 1990; Sampson et al., 2010:63; Farke et al., 2011:43)

118. Squamosal, shape of temporal process:
(0) – simple
(1) – deeply bifurcate around temporal process of postorbital
(Modified from Makovicky and Norell, 2006:37)

119. Squamosal, parietosquamosal frill posterior expansion:
(0) – very slight, short shelf subequal to posterior extent of occipital condyle
(1) – intermediate shelf, expands posterior to the occipital condyle
(2) – extensive shelf, squamosal and parietal expanded well beyond the posterior extent of the quadrates forming true frill
(New Character; modified to some extent from Makovicky and Norell, 2006:52)

120. Squamosal, shape of posterior expansion:
(0) – sub-rectangular in outline
(1) – fan-shaped, sub-triangular
(2) – fan-shaped, sub-triangular with an extended posterior ramus
(3) – sickle-shaped, posteriorly narrowed

(Modified from Makovicky and Norell, 2006:36; Sampson et al., 2010:59, Farke et al., 2011:41)

121. Squamosal, posterior otic notch formed by the posterioventral expansion of the squamosal:
(0) – absent
(1) – present
(New Character)

122. Squamosal, length relative to parietal on frill:
(0) – squamosal as long as the parietal and forms part of the posterior margin of the frill
(1) – squamosal shorter than parietal with part of the lateral portion of the frill made up of parietal
(Modified from Sampson et al., 2010:60-61)

123. Squamosal, medial lamina forming the posteriolateral floor of dorsotemporal fossa creating a pocket in the squamosal in dorsal view:
(0) – absent
(1) – present
(Modified from Dodson, 1986; Sampson et al., 2010:60, Farke et al., 2011:42)

124. Squamosal, posterior edge of squamosal is stepped so that lateral portion extends further posteriorly in dorsal view creating a "stepped margin":
(0) – absent
(1) – present
(Modified from Dodson, 1986; Sampson et al., 2010:60)

125. Squamosal, extent of step "stepped margin":
(0) – slight step where posterior expansion of step is less than the dorsoventral thickness of the pocket
(1) – posteriorly expanded to greater than twice the dorsoventral thickness of the pocket
(New Character)

126. Squamosal, thickened, rounded swelling along medial margin:
(0) – absent, lateral surface of squamosal flat to slightly convex
(1) – present, lateral surface of squamosal slightly concave
(Forster, 1990; Farke, 2006; Sampson et al., 2010:64, Farke et al., 2011:44)

127. Squamosals, orientation of temporal bars:
(0) – parallel
(1) – posteriorly divergent
(Modified from Makovicky and Norell, 2006:39)

128. Squamosal, shape of dorsal edge of temporal bar as it meets posterior frill margin:
(0) – curves medially at the posterior end, arcing confluently into posterior frill margin
(1) – frill at acute angle
(Modified from Makovicky and Norell, 2006:53)

129. Squamosals, orientation of posterior edge:
(0) – angled anteromedially
(1) – angled posteromedially, squamosal contributing lateral portion of frill margin
(Modified from Makovicky and Norell, 2006:38)

130. Squamosal, ridge along the posterioventral edge of squamosal:
(0) – absent
(1) – present
(Modified from Farke et al., 2014:141)

131. Squamosal, oblique dorsal ridge or bumps forming ridge along the dorsal surface of the squamosal:
(0) – absent
(1) – present
(2) – pronounced
(Modified from Evans and Ryan, 2015:98)

Parietal

132. Parietal, anterior extent on dorsum of skull relative to occipital condyle:
(0) – anterior end of parietal located well in front of occipital condyle
(1) – anterior end of parietal lies directly over occipital condyle

(Sampson et al., 2010:57; Farke et al., 2011:40)

133. Parietal, shape of skull roof:
(0) – flat or gently convex
(1) – sharp midline crest
(Modified from Farke et al., 2014:138)

134. Parietosquamosal contact, shape in dorsal view:
(0) – lancelet or straight
(1) – curved, medially concave
(Modified from Forster 1990:66; Sampson et al., 2010:57; Farke et al., 2011:45)

135. Parietal, concave median embayment on posterior margin:
(0) – absent
(1) – present
(Makovicky and Norell, 2006:55; Sampson et al., 2010:66; Farke et al., 2011:46)

136. Parietal, shape of concave median embayment on posterior edge of parietal:
(0) – shallow, may include the whole back of the frill
(1) – restricted to center of margin, incipient
(2) – restricted to center of margin, deep notch-like
(Modified from Forster et al., 1993:9; Forster, 1990:83; Sampson et al., 2010:67, Farke et al., 2011:47)

137. Parietal, location of posterior-most point of parietal:
(0) – on midline
(1) – between midline and lateral-most corner
(2) – at lateral-most corner adjacent to squamosal
(Modified from Holmes et al., 2001:18; Sampson et al., 2010:68)

138. Parietosquamosal frill, elongation into frill:
(0) – absent
(1) – present elongation of parietal into frill

139. Parietosquamosal frill, length relative to basal skull length:
(0) – short, 60% or less
(1) – elongate, more than 70%
(Modified from Hatcher et al., 1907; Lehman, 1990; Forster, 1990: XX; Sampson et al., 2010:69)

140. Parietosquamosal frill, location of maximum transverse width true frill:
(0) – anteriorly, in association with proximal third of frill
(1) – widest part in middle, frill round
(2) – lateral edges roughly parallel
(3) – posteriorly
(Modified from Holmes et al., 2001:17; Sampson et al., 2010:70)

141. Parietal, parietal sulci:
(0) – absent
(1) – present or secondarily roofed
(Marsh, 1892; Sampson et al., 2010:71)

142. Parietal, overall shape:
(0) – nearly straight along midline in lateral view and gently arched from side to side
(1) – "saddle-shaped," dorsally concave in lateral view with upturned posterior margin, and arched strongly from side to side
(Sampson et al., 2010:72)

143. Parietal, sharp median crest:
(0) – present as ridge or line
(1) – absent
(Sampson et al., 1997; Sampson et al., 2010:75; Farke et al., 2011:49)

144. Parietal fenestra:
(0) – absent, no fenestra
(1) – present
(Modified from Forster, 1990: XX; Makovicky and Norell, 2006:54; Sampson et al., 2010:73)

145. **Parietal, rim on medial margin of dorsotemporal fenestra:**
(0) – absent
(1) – present, well-defined but narrow laterally projecting rim defines medial margin of fenestra
(2) – present, well-defined and wide laterally projecting rim defines medial margin of fenestra
(Modified from Forster, 1990; Sampson et al., 2010:74; Farke et al., 2011:48)

146. **Parietal, anteroposterior thickness of transverse bar at narrowest point:**
(0) – narrow and straplike, less than 10% total parietal length
(1) – broad, 20% or more of total parietal length
(Modified from Forster et al., 1993:3; Sampson et al., 2010:76; Farke et al., 2011:50)

147. **Parietal, cross-sectional shape of transverse bar:**
(0) – flat
(1) – round
(New Character)

148. **Parietal, relative anteroposterior depth of transverse bar:**
(0) – subequal medial to lateral
(1) – tapering medially
(Sampson et al., 2010:77)

149. **Parietal, cross-sectional shape of median bar:**
(0) – subtriangular, tapers laterally
(1) – round to lenticular
(2) – v-shaped without thickening
(Modified from Holmes et al., 2001:24; Sampson et al., 2010:78)

150. **Parietal, median bar, transverse width:**
(0) – narrow and straplike, transverse width less than 10% total parietal length
(1) – relatively wide, transverse width 15% or more of total parietal length
(Holmes et al., 2001:23; Sampson et al., 2010:79; Farke et al., 2011:51)

151. **Parietal fenestra, orientation:**
(0) – long axis directed transversely
(1) – long axis directed axially
(2) – axial and transverse axes equal
(Modified from Forster, 1990; Sampson et al., 2010:80)

152. **Parietal fenestra size, maximum proximodistal diameter:**
(0) – tiny, 35% or less total parietal length
(1) – large, greater than 45% total parietal length
(Modified from Forster et al., 1993:8; Sampson et al., 2010:81)

Epiossifications

153. **Marginal dermal ossifications on parietal and squamosal capping marginal undulations:**
(0) – absent
(1) – present
(Modified from Forster 1990:91 & 92; Makovicky and Norell, 2006:56; Sampson et al., 2010:84; Farke et al., 2011:53)

154. **Parietosquamosal frill, imbrication of undulations:**
(0) – absent
(1) – present
(Sampson et al, 1997; Dodson et al., 2004:34; Sampson et al., 2010:83; Farke et al., 2011:52)

Episquamosals

155. **Episquamosal, shape:**
(0) – crescentic or ellipsoidal, often with constricted base
(1) – triangular/peaked often elongated
(Dodson et al., 2004:45; Sampson et al., 2010 87; Farke et al., 2011:54)

156. **Episquamosals, large gaps between epiquamosals:**

(0) – large gaps present between episquamosals
(1) – absent, episquamosals nearly touch one another along squamosal margin
(New Character)

157. Episquamosal, size variation:
(0) – all subequal in size
(1) – marked asymmetry in size
(Modified from Sampson et al., 2010:85, 86)

158. Episquamosal, asymmetry:
(0) – only S1 enlarged
(1) – S1 and S2 enlarged
(2) – anterior-most episquamosal is largest
(New Character)

159. Length and height of all episquamosals:
(0) – sub-equilateral delta or crescentic shaped
(1) – all epiquamosals long with high epiquamosal length to height ratio
(Sampson et al., 2010:85)

160. Number of episquamosals on squamosal:
(0) – 4 or less
(1) – five
(2) – six or seven episquamosals
(3) – eight or more episquamosals
(Modified from Farke et al., 2011:55)

Epiparietosquamosals

161. Marginal ossification crossing parietal-squamosal contact (location EPS):
(0) – absent
(1) – present
(Modified from Dodson et al., 2004:43; Sampson et al., 2010:91; Farke et al., 2011:56)

Epiparietals

162. Epiparietal, fusion to adjacent epiparietals at base:
(0) – absent
(1) – present
(Modified from Holmes et al., 2001:29)

163. Epiparietal, number per side not counting midline p0 or eps:
(0) – five or more
(1) – three
(2) – only one
(Modified from Holmes et al., 2001:28; Farke et al., 2011:57)

164. Epiparietal, presence on midline margin (P0 locus):
(0) – absent
(1) – present
(New Character)

165. Epiparietal, shape of P0:
(0) – ellipsoidal or crescentic, with constricted base
(1) – triangular, peaked
(New Character)

166. Epiparietal, epiparietal ep1 coalesced:
(0) – absent
(1) – present
(New character)

167. Epiparietal, shape of general epiparietals:
(0) – ellipsoidal or crescentic, often with constricted base

(1) – triangular, peaked

168. Parietal, nodes or raised bumps on dorsal surface of posterior margin in between epiparietal loci (dorsoparietal processes:

(0) – absent
(1) – present
(New Character)

169. Epiparietal, multiple raised undulations on proximal midline ramus of parietal:

(0) – absent
(1) – present
(New charcter)

Epiparietal Homology Hypothesis C: Clayton and Loewen *(18 characters)*

170. Epiparietal, ep1, presence:

(0) – present
(1) – absent, or vestigial
Note: we interperet the loss of ep1 in *Rubeosaurus, Einiosaurus, Achelosaurus* and *Pachyrhinosaurus canadensis* as evidenced by vestigual ep1s present in some specimens of *Pachyrhinosaurus lacustai* and its presence in *Pachyrhinosaurus perotorum*.
(New Character)

171. Epiparietal, ep1 length:

(0) – short
(1) – elongate, forms spike
(New Character)

172. Epiparietal, ep1 shape:

(0) – flattened
(1) – round or oval cross-section
(New Character)

173. Epiparietal, ep1 orientation relative to plane of frill:

(0) – in the plane of frill
(1) – curled dorsally or anterodorsally
(New Character)

174. Epiparietal, ep1 lateral curvature:

(0) – in plane of frill
(1) – curved laterally or medially
(New Character)

175. Epiparietal, ep1, curved elements:

(0) – curve laterally
(1) – curve medially
(New Character)

176. Epiparietal, ep2, presence:

(0) – absent
(1) – present
(New Character)

177. Epiparietal, shape of ep2:

(0) – flat
(1) – round
(New Character)

178. Epiparietal, ep2 elongation:

(0) – ep2 is similar to other epiparietals
(1) – elongated into either a triangle, hook or spike
(New Character)

179. Epiparietal, elongated ep2:

(0) – hook about 3 times longer than wide
(1) – spike, elongated more than 6 times longer than wide

(New Character)

180. **Epiparietal, ep2 elongated epiparietal shape:**
(0) – greatly flattened dorsoventrally
(1) – round or oval cross-section
(New Character)

181. **Epiparietal, ep2 orientation:**
(0) – in plane of the frill
(1) – curled dorsally or anterodorsally
(New Character)

182. **Epiparietal, ep2 mediolateral curvature:**
(0) – straight
(1) – curled or oriented medially or laterally

183. **Epiparietal, ep3, presence:**
(0) – absent
(1) – present
(New Character)

184. **Epiparietal, elongated ep3:**
(0) – flat
(1) – round
(New Character)

185. **Epiparietal, ep3 length:**
(0) – short, small and crescentic
(1) – elongated into either a triangle, hook or spike
(New Character)

186. **Epiparietal, elongated ep3:**
(0) – elongated triangular process
(1) – elongated more than 3 times longer than wide
(New Character)

187. **Epiparietal, ep4, presence:**
(0) – absent
(1) – present
(New Character)

188. **Epiparietal, ep4 length:**
(0) – short, small and crescentic
(1) – elongated into either a triangle, hook or spike
(New Character)

189. **Epiparietal, elongated ep4:**
(0) – elongated triangular process
(1) – elongated more than 3 times longer than wide
(New Character)

190. **Epiparietal, ep5, presence:**
(0) – absent
(1) – present
(New Character)

191. **Epiparietal, ep5 shape:**
(0) – short, retains general shape of unmodified epiparietals
(1) – developed into spike
(New Character)

192. **Epiparietal, shape of the most proximal epiparietal (usually ep7):**
(0) – no ep5, ep6 or ep7
(1) – small and crescentic
(2) – triangular process
(3) – developed into triangular flattened spike

Braincase

193. **Supraoccipital, contribution to foramen magnum:**
(0) – forms dorsal margin of foramen magnum
(1) – eliminated from margin by exoccipital-exoccipital contact on midline
(Brown & Schlaikjer, 1940a; Forster 1990:63; Makovicky and Norell, 2006:65; Farke et al., 2011:69)

194. **Supraoccipital, ventrolateral processes:**
(0) – absent
(1) – present
(Brown & Schlaikjer, 1940a)

195. **Supraoccipital, ventrolateral processes, dorsolateral ridge between midline supraoccipital ridge and top of exoccipital wing:**
(0) – absent
(1) – present

196. **Supraoccipital, ventrolateral processes, dorsolateral ridge orientation:**
(0) – oriented at roughly 45 degrees
(1) – originate at 45 degrees and then extend dorsally parallel to the midline ridge

197. **Supraoccipital, deep fossa between dorsolateral strut and exoccipital:**
(0) – no fossa
(1) – shallow depression
(2) – deep fossa that forms dorsomedially inclined strut that joins dorsolateral strut

198. **Supraoccipital orientation:**
(0) – anteriorly inclined relative to basioccipital
(1) – posteriorly inclined or in same plane as posterior face of basioccipital
(Makovicky & Norell, 2006:66)

199. **Supraoccipital shape:**
(0) – tall, triangular
(1) – or wider than tall, trapezoid
(2) – or square
(Makovicky & Norell, 2006:67)

200. **Supraoccipital, midline vertical ridge:**
(0) – absent
(1) – present
(new character)

201. **Supraoccipital, midline vertical ridge, form:**
(0) – rounded ridge
(1) – ridge forms fin
(new character)

202. **Supraoccipital, shape of posterior surface:**
(0) – flat, convex, or with midline ridge
(1) – midline depression along base of midline ridge
(Modified from Farke et al., 2014:142)

203. **Basioccipital, contribution to occipital condyle:**
(0) – forms more than 2/3 of occipital condyle
(1) – forms less than 1/3 of the occipital condyle
(Brown & Schlaikjer, 1940a)

204. **Basioccipital, participates in foramen magnum:**
(0) – present
(1) – or basioccipital is excluded from foramen magnum
(Makovicky & Norell, 2006:57)

205. **Basioccipital, subcondylar region form:**
(0) – raised ridge ventral to occipital condyle
(1) – flat or rounded
(new character)

206. **Basioccipital, presence of fossa in raised subcondylar region:**

(0) – absent
(1) – fossa is present ventral to occipital condyle
(new character)

207. **Basioccipital, subcondylar region, ventral process lateral to midline vertical ridge:**
(0) – present between ventral process and basitubera
(1) – absent
(new character)

208. **Basioccipital, contribution to basal tubera:**
(0) – excluded from basal tubera by basisphenoid and limited to occipital midline
(1) – basioccipital contributes to basal tubera present
(Modified from Makovicky and Norell, 2006:58)

209. **Basal tubera, position:**
(0) – flat, in plane with basioccipital plate
(1) – everted posterolaterally, forming lip beneath occipital condyle
(Modified from Makovicky and Norell, 2006:60)

210. **Basal tubera, fossa on posterior surface:**
(0) – absent
(1) – present
(New Character)

211. **Basisphenoid, notch between posteroverntral edge of basisphenoid and base of basipterygoid process:**
(0) – deep
(1) – shallow and base of basipterygoid process close to basioccipital tubera
(Modified from Makovicky and Norell, 2006:61)

212. **Foramen magnum, visible in posterior view:**
(0) – visible in posterior view
(1) – dorsally oriented and obscured by occipital condyle
(new character)

213. **Olfactory nerve (I), frontal contribution to exit from braincase:**
(0) – present, frontal forms roof of olfactory tract
(1) – absent, olfactory tract enclosed entirely by ossification of the laterspenoid.
(Forster, 1990)

214. **Exoccipital, exits for cranial nerves in exoccipital:**
(0) – three foramina
(1) – two foramina
(Brown & Schlaikjer, 1940a; Forster, 1990; Makovicky and Norell, 2006:62)

215. **Exoccipital–quadrate:**
(0) – completely separated by ventral flange of squamosal
(1) – or in contact
(Makovicky & Norell, 2006:63)

216. **Paroccipital process, dorsoventral distal expansion:**
(0) – distal process only slightly expanded
(1) – distal process expanded to at least .8 two times the depth at its narrowest point
(Brown & Schlaikjer, 1940a; Makovicky and Norell, 2006:64)

Pterygoid

217. **Pterygoid, contacts maxilla posterior to the tooth row:**
(0) – absent
(1) – present
(Makovicky & Norell, 2006:47)

218. **Pterygoid, mandibular process:**
(0) – long, extending well below maxillary tooth row
(1) – short
(Modified after Makovicky & Norell, 2006:49)

219. **Pterygoid, mandibular process makeup:**
(0) – jointly formed by pterygoid and ectopterygoid
(1) – formed only by pterygoid
(Modified from Makovicky and Norell, 2006:50)

220. **Pterygoid, ventral ridge on mandibular process of pterygoid defining Eustachian canal:**
(0) – absent
(1) – present
(Makovicky and Norell, 2006:46)

221. **Pterygoid, prominent posterior midline process:**
(0) – absent
(1) – present
(Modified from Makovicky and Norell, 2006:48)

Ectopterygoid

222. **Ectopterygoid contacts jugal:**
(0) – present
(1) – or ectopterygoid reduced and restricted to contact with maxilla
(Brown & Schlaikjer, 1940a; Makovicky & Norell, 2006:44)

223. **Ectopterygoid visible in palatal view:**
(0) – exposed
(1) – or reduced and concealed in palatal view
(Makovicky & Norell, 2006:43)

Palatine and vomer

224. **Secondary palate, relative contribution of maxilla:**
(0) – maxilla forms at least 45% of the length of the secondary palate
(1) – maxilla contributes only to the posterior portion, forms 30% or less of secondary palate
(Brown & Schlaikjer, 1940a)

225. **Palatine, shape and relationship to maxilla:**
(0) – palatine contacts nearly the entire medial surface of the maxilla, restricting size of choanae, anterodorsal process embraces posterior end of vomer
(1) – palatine contacts only the posterior one-third of medial surface of maxilla, contact with vomer lost, choanae enlarged
(Osmólska, 1986)

226. **Elongate parasagittal process of the palatine:**
(0) – absent
(1) – present
(Makovicky & Norell, 2006:42)

227. **Vomer, relationship to maxillae on secondary palate:**
(0) – vomers insert between maxillae at the rear of the secondary palate
(1) – vomers meet posterior margin of maxillae on secondary palate, do not insert between maxillae, so that the maxillae contact each other anterior to the choanae in palatal view and obscure the tip of the vomer
(Modified from Makovicky & Norell, 2006:21)

Quadrate

228. **Quadrate, shape of the anterior quadrate shaft in lateral view:**
(0) – convex
(1) – straight
(Modified from Makovicky and Norell, 2006:41)

Mandible

229. **Lower jaw, level of mandibular articulation:**

(0) – at or slightly below occlusal surface of tooth row
(1) – depressed well below level of occlusal surface of tooth row
(Ostrom, 1964; Forster, 1990; Sampson et al., 2010:112)

230. External mandibular fenestra:
(0) – present
(1) – absent
(new character)

231. Predentary/dentary anteroposterior ratio:
(0) – less than two thirds of dentary length
(1) – equal to or more than two-thirds of dentary length
(Makovicky & Norell, 2006:70; Sampson et al., 2010:113)

232. Rostral and predentary shape in occlusal view:
(0) – broad and rounded anteriorly, “U” shaped
(1) – acute, “V”-shaped
(new character)

233. Predentary, shape of dorsal surface:
(0) – shallow or flat
(1) – scooplike
(Modified from Makovicky and Norell, 2006:68)

234. Predentary, dentary processes:
(0) – ventral processes much longer than abbreviated dorsal processes
(1) – dorsal and ventral processes elongate and subequal in length
(Sampson et al., 2010:114; Farke et al., 2011:70)

235. Predentary, ventral dentary process shape:
(0) – wedge shaped and mediolaterally broad
(1) – splint shaped
(Modified from Makovicky and Norell, 2006:69)

236. Predentary, occlusal margin:
(0) – mediolaterally thin
(1) – thickened labiolingually
(new character)

237. Predentary buccal margin:
(0) – with round beveled edge
(1) – or with grooved, triturating edge
(Makovicky & Norell, 2006:71)

238. Predentary, orientation of triturating surface:
(0) – nearly horizontal
(1) – inclined steeply laterally
(2) – inclined medially
(Lehman, 1990; Forster, 1990; Dodson et al., 2004:57; Farke et al., 2011:71)

239. Predentary, contact of the lateral process with dentary:
(0) – smooth
(1) – grooved dorsally
(2) – bears large pit
(Modified from Makovicky and Norell, 2006:72)

240. Dentary, symphyseal area:
(0) – small excluding splenial
(1) – large, forming strong immobile bond with participation of splenial
(Modified from Makovicky and Norell, 2006:73)

241. Dentary, presence of diastema at anterior end:
(0) – absent
(1) – present
(Modified from Makovicky and Norell, 2006:74)

242. **Dentary, presence of ventral flange:**
(0) – absent
(1) – present along ventral edge
(Modified from Makovicky and Norell, 2006:76)

243. **Dentary, lateral ridge confluent with cutting surface of predentary:**
(0) – present
(1) – absent
(Farke et al., 2011:72)

244. **Dentary, prominent medial expansion of the central mandible in the middle of the tooth row formed by wide Meckelian groove separating tooth-bearing part of the jaw from external surface:**
(0) – absent
(1) – present
(Modified from Makovicky and Norell, 2006:77)

245. **Dentary, shape of area lateral to tooth row:**
(0) – smooth
(1) – rugose and sculpted
(Modified from Makovicky and Norell, 2006:78)

246. **Dentary anteroventral fossa below lateral ridge:**
(0) – absent
(1) – present
(new character)

247. **Dentary anteroventral fossa below lateral ridge size:**
(0) – deep
(1) – weakly present
(new character)

248. **Dentary, shape of ventral margin in adults:**
(0) – strongly convexly bowed
(1) – straight
(Brown and Schlaikjer, 1940a; Forster, 1990:73; Makovicky and Norell, 2006:75; Sampson et al., 2010:117)

249. **Dentary, posterior extent of tooth row:**
(0) – terminates anterior to the center of the coronoid process
(1) – terminates even with the coronoid process
(2) – terminates posterior to the coronoid process
(Brown & Schlaikjer, 1940a; Chinnery and Weishampel 1998; modified from Makovicky and Norell, 2006:82; Sampson et al., 2010:118; Farke et al., 2011:73)

250. **Dentary, overall shape of coronoid process:**
(0) – short, with gently convex apex, base of ascending ramus anteroposteriorly expanded
(1) – tall, expanded at apex into anteriorly projecting hook, base of ascending ramus anteroposteriorly restricted
(Lull, 1933; Sampson et al., 2010:119)

251. **Dentary, shape of dorsal end of coronoid process:**
(0) – rounded
(1) – anterior expansion
(Modified from Makovicky and Norell, 2006:81)

252. **Dentary, coronoid process notch along posterior edge of dentary:**
(0) – absent
(1) – present
(Modified from Farke et al., 2014:145)

253. **Dentary, width of coronoid process notch:**
(0) – wide
(1) – constricted notch
(Modified from Farke et al., 2014:146)

254. **Dentary, separation of body from ascending ramus of coronoid process:**
(0) – absent
(1) – present
(Granger & Gregory, 1923; Sampson et al., 2010:120)

255. **Splenial, shape:**
(0) – nearly as deep as the body of the dentary, does not contact articular, angular exposed in medial view
(1) – shallow, contacts articular, covers angular in medial view
(Brown & Schlaikjer, 1940a, 1940b; Sampson et al., 2010:121)

256. **Posterior end of splenial:**
(0) – simple or with shallow dent
(1) – or with bifid overlap of angular
(Makovicky & Norell, 2006:80)

257. **Coronoid bone, size:**
(0) – large and lobate, with expanded dorsal end much deeper than ventral that inserts between the dentary and splenial to contact, contacts the splenial
(1) – small and strap-like with subequal depth throughout, no contact with splenial
(New Character derived from Brown & Schlaikjer, 1940a, 1940b; modified from Makovicky and Norell, 2006:83)

258. **Angular, exposure in lateral view:**
(0) – extensive
(1) – greatly restricted
(New Character derived from Brown & Schlaikjer, 1940a)

259. **Angular, shape of lateral surface:**
(0) – or flat or slightly convex
(1) – angular bears a raised emargination along posteroventral margin of mandible, lateral surface distinctly concave
(Modified after Makovicky & Norell, 2006:88)

260. **Angular, texture of lateral surface:**
(0) – smooth
(1) – strongly sculptured
(Modified from Farke et al., 2014:135)

261. **Angular, presence of one or more small lateral tubercles along ventral rib below glenoid articulation:**
(0) – absent
(1) – present
(Modified from Makovicky and Norell, 2006:87)

262. **Surangular dorsally projecting ridge on lateral margin:**
(0) – present
(1) – absent
(Makovicky & Norell, 2006:84)

263. **Surangular, lateral surface:**
(0) – flat or only weakly convex
(1) – with pronounced laterally convex curvature (in the transverse plane) between the coronoid process and glenoid region
(Modified from Makovicky and Norell, 2006:85)

264. **Surangular, lateral process below glenoid:**
(0) – absent
(1) – present; surangular knob
(Farke et al., 2014:139)

265. **Surangular, presence of tab forming lateral wall to glenoid cotyle:**
(0) – absent
(1) – present
(Modified from Makovicky and Norell, 2006:86)

266. Angular–surangular–dentary contact:
(0) – triradiate
(1) – surangular with long ventral process overlapping angular, dentary-surangular and angular-surangular sutures form acute angle on lateral face of mandible
(Makovicky & Norell, 2006:89)

267. Prearticular-dentary contact:
(0) – absent
(1) – present
(Brown & Schlaikjer, 1940a, 1940b; Makovicky and Norell, 2006:79; Sampson et al., 2010:122)

268. Articular, size and contribution to mandibular glenoid:
(0) – articular forms only the medial 1/3 of the articular surface, extends posteriorly as far as the angular
(1) – articular enlarged, forms half of the articular surface, extends behind angular to form rear of lower jaw
(New Character derived from Brown & Schlaikjer, 1940a)

269. Articular, mandibular glenoid in dorsal view:
(0) – narrow and flush with medial margin of surangular flange
(1) – glenoid region medially expanded and forming lingual process
(Modified from Makovicky and Norell, 2006:90)

270. Glenoid, surface of prearticular and articular below glenoid:
(0) – smooth
(1) – wide, semicircular ventral process near medial face of glenoid
(Modified from Makovicky and Norell, 2006:91)

271. Articular, retroarticular process length:
(0) – long
(1) – short or absent
(Modified from Makovicky and Norell, 2006:92)

Dentition

272. Tooth, number of roots:
(0) – one
(1) – two
(Forster 1990:34; Makovicky and Norell, 2006:95; Sampson et al., 2010:123; Farke et al., 2011:74)

273. Teeth, presence of median primary ridge:
(0) – absent
(1) – very weak and wide median ridge on at least some maxillary teeth
(2) – all maxillary and dentary teeth with distinct primary ridge
(Modified from Makovicky and Norell, 2006:98)

274. Teeth, presence of cingulum:
(0) – absent
(1) – present
(Modified from Makovicky and Norell, 2006:100)

275. Teeth, relationship of the base of primary ridge and the cingulum:
(0) – confluent with the cingulum on maxillary teeth
(1) – base of primary ridge set back from cingulum; forms a continuous ridge at the crown base
(Modified from Makovicky and Norell, 2006:99)

276. Teeth, enamel on lingual and buccal surfaces of teeth:
(0) – covered with enamel
(1) – enamel restricted to lateral side of maxillary and medial side of dentary teeth
(Modified from Makovicky and Norell, 2006:102)

277. Tooth crown height in mid tooth row position:
(0) – subequal to tooth crown width
(1) – taller than wide

(New Character)

278. **Teeth, crown shape in lateral view:**
(0) – radiate or pennate
(1) – ovate
(Modified from Makovicky and Norell, 2006:106)

279. **Cheek teeth, shape of roots:**
(0) – with cylindrical roots
(1) – roots with mesial and distal faces flattened to slightly grooved
(Modified from Makovicky and Norell, 2006:105)

280. **Teeth, cheek teeth spacing:**
(0) – spaced
(1) – closely appressed
(Makovicky & Norell, 2006:96)

281. **Teeth, occlusion:**
(0) – occlude at an oblique angle
(1) – at a vertical angle
(2) – at a vertical angle, but dentary teeth have a horizontal shelf on the labial face
(Makovicky & Norell, 2006:97)

282. **Tooth, number of replacements per alveolus:**
(0) – one or two replacement teeth
(1) – three or more replacement teeth
(Sereno 1999:137; Makovicky and Norell, 2006:101; Farke et al., 2011:76)

283. **Tooth magazine, case-like alveolar slots for vertical tooth families formed by spongy bone:**
(0) – absent
(1) – present
(Brown & Schlaijker, 1940a)

284. **Premaxillary teeth:**
(0) – absent
(1) – present
(New Character)

285. **Premaxillary teeth:**
(0) – three or more teeth in premaxilla
(1) – or two teeth in premaxilla
(2) – or 1 tooth in premaxilla
(Makovicky & Norell, 2006:93)

286. **Premaxillary teeth morphology:**
(0) – with carinae and in some cases serrations
(1) – premaxillary teeth peglike, crown without carinae
(Makovicky & Norell, 2006:94)

287. **Maxillary teeth, number of alveoli in maxilla:**
(0) – 11 or less
(1) – more than 12 and less than 14
(2) – more than 15 and less than 18
(3) – more than 20 and less than 29
(4) – more than 30
(New Character)

288. **Dentary teeth, number of alveoli in dentary:**
(0) – 11 or less
(1) – more than 12 and less than 18
(2) – more than 19 and less than 29
(3) – more than 30
(Modified from Makovicky & Norell, 2006:104; Farke et al., 2011:75)

289. **Dentary teeth, shape of tooth crowns:**

(0) – continuous, smooth root crown transition
(1) – bulbous expansion at root-crown transition on labial side of tooth, sometimes worn to form notch or shelf
(Modified from Makovicky and Norell, 2006:103)

Axial Skeleton

290. Atlantal intercentrum, shape:
(0) – semicircular
(1) – disc shaped
(Modified from Makovicky and Norell, 2006:107)

291. Atlantal intercentrum, fusion to odontoid:
(0) – absent
(1) – present
(Modified from Makovicky and Norell, 2006:108)

292. Atlantal intercentrum, fusion to atlantal neuropophyses:
(0) – absent
(1) – present
(Modified from Makovicky and Norell, 2006:109)

293. Cervical vertebrae, formation of syncervical:
(0) – absent
(1) – present
(Modified from Forster 1990:122; Sampson et al., 2010:127; Farke et al., 2011:77)

294. Cervical vertebrae, form of syncervical:
(0) – C1-3fused or tightly articulated, atlantal intercentrum present as a ventrally placed, wedge-like bone
(1) – C1-3 firmly fused, atlantal intercentrum (= hypocentrum) forms a complete ring
(Modified from Forster 1990:122; Sampson et al., 2010:127; Farke et al., 2011:77)

295. Axis, neural spine shape and orientation
(0) – blade-like and nearly vertical, overhangs only anterior-most portion of C3
(1) – blade-like morphology lost, spine steeply angled to reach posterior margin of C3
(Brown & Schlaikjer, 1940a; Sereno 1999:141; modified from Makovicky and Norell, 2006:110,111; Sampson et al., 128: Farke et al., 2011:78):

296. Atlantal rib:
(0) – present
(1) – absent
(Brown & Schlaikjer, 1940a; Sampson et al., 2010:129; Farke et al., 2011:79)

297. Cervical centra, presence of ventral keels:
(0) – ventral keels present
(1) – some or all postaxial centra without keels
(Modified from Farke et al 2014:147)

298. Cervical rib shape:
(0) – L-shaped
(1) – y-shaped
(2) – x-shaped accessory dorsal process present
(New Character)

299. Mid-dorsal neural spine height:
(0) – subequal in height to centrum height
(1) – 2 times the centrum height
(2) – three times centrum height
(New Character)

300. Dorsal vertebrae, zygapophyseal articular surfaces shape:
(0) – with flat articulations on zygapophyses
(1) – tongue and grooves articulations on zygapophyses

(Makovicky & Norell, 2006:113)

301. Dorsal vertebrae, shape of centra:
(0) – relatively axially elongate, lengths exceeds width length/height of centra XX or more
(1) – axially shortened, width exceeds length length/height of centra XX or less
(Modified from Sampson et al., 2010:130; Farke et al., 2011:80)

302. Dorsal vertebrae, elongation of tallest neural arches:
(0) – arch shorter than centrum
(1) – present, arch height taller than centrum
(New Character)

303. Dorsal vertebrae, posteriormost dorsals, zygopohyseal facets horizontal, zygopophyses coalesce:
(0) – absent
(1) – present
(New Character)

304. Sacrum, number of vertebrae:
(0) – five
(1) – six
(2) – seven
(3) – eight or more
(Modified from Makovicky and Norell, 2006:114)

305. Sacrum, longitudinal sulcus on ventral surface:
(0) – absent
(1) – present restricted to caudal sacral vertebrae
(2) – deep across nearly all sacral vertebrae
(Modified from Lambe, 1915; Lehman, 1989, 1990; Sampson et al., 2010:131; Farke et al., 2011:81)

306. Sacrum, shape in dorsal view:
(0) – rectangular
(1) – suboval, mid-sacral ribs longest
(Modified from Makovicky and Norell, 2006:115)

307. Sacrum, ventral expansion of 1st sacral rib to attach along entire lateral side of vertebra:
(0) – absent
(1) – present
(New Character derived from Lull, 1933; Lehman, 1990)

308. Anterior caudal neural spines, height:
(0) – short and anteriorly inclined
(1) – tall and erect
(Makovicky & Norell, 2006:116)

309. Mid caudal neural spines, height:
(0) – less than or equal to 2
(1) – 2.1 to 3 times
(2) – 3.1 to 4 times
(3) – more than 4.1 times
(Modified from Farke et al., 2014:140)

310. Distal caudals, tail terminates with:
(0) – series of cylindrical caudals that are devoid of neural spines and chevrons
(1) – neural spines and chevrons persist virtually to the end of tail
(Modified from Makovicky and Norell, 2006:117)

311. Caudal chevrons, shape of distal caudal chevrons:
(0) – lobate expanded shape
(1) – rodlike
(Modified from Makovicky and Norell, 2006:118)

Pectoral Girdle and Limb

312. Scapula, shape in lateral view:

(0) – distinctly curved in lateral view
(1) – relatively straight
(Makovicky & Norell, 2006:120)

313. Scapula, position of blade relative to glenoid:
(0) – at acute angle
(1) – almost perpendicular to glenoid
(Modified from Makovicky and Norell, 2006:121)

314. Scapula, relative contribution to glenoid fossa:
(0) – scapula and coracoid contribute equally
(1) – scapula contributes well over half of the glenoid
(Brown & Schlaikjer, 1940a; Sereno 1999:145; Farke et al., 2011:82)

315. Glenoid fossa, size and shape:
(0) – glenoid relatively short, unexpanded mediolaterally
(1) – glenoid expanded anteroposteriorly and expanded medially
(Chinnery, 2004)

316. Glenoid fossa, position of humeral head:
(0) – positioned in the lateral portion of the glenoid fossa
(1) – positioned medially in the glenoid fossa
(New Character)

317. Scapular ridge or scapular spine:
(0) – absent
(1) – present
(New Character)

318. Scapula, orientation of scapular spine:
(0) – runs obliquely across blade
(1) – runs longitudinally along blade
(Sampson et al., 2010: 133)

319. Scapula, size of acromion process:
(0) – small, restricted to proximal end
(1) – enlarged, extends distal to the glenoid fossa
(Modified from Makovicky and Norell, 2006:123; Maidment and Barrett, 2011:)

320. Coracoid, shape of anterior blade:
(0) – smooth, arcuate anterior portion
(1) – bearing large anterolateral ridge near confluence of anterior and ventral margins
(Modified from Makovicky and Norell, 2006:122)

321. Coracoid, medial opening of coracoid foramen connected to coraco-scapular suture by sulcus:
(0) – absent
(1) – present
(New Character)

322. Coracoid, concave inflection between coraco-scapular suture and anterior margin of coracoid:
(0) – absent
(1) – present
(New Character)

323. Clavicle:
(0) – present
(1) – absent
(Sereno 1999:147; Makovicky & Norell, 2006:119; Farke et al., 2011:84)

324. Humerus, length of deltopectoral crest in adult:
(0) – short, less than 40% total humeral length
(1) – elongate, subequal to or greater than 45% of the total humeral length
(Chinnery, 2001, 2004)

325. Humerus, shape of margin of deltopectoral crest:
(0) – concave
(1) – straight to convex

(Maidment and Barrett, 2011)

326. Humerus, triceps fossa on posteriolateral deltopectoral crest:
(0) – shallow with poorly defined margins
(1) – deep with well-defined margins
(Maidment and Barrett, 2011)

327. Humerus, position of foramen on posteriomedial humerus relative to apex of deltopectoral crest:
(0) – level with
(1) – proximal to
(Maidment and Barrett, 2011)

328. Humerus, orientation of deltopectoral crest:
(0) – projects anteriorly
(1) – projects anterolateraly
(Maidment and Barrett, 2011)

329. Radius, lateral and medial tuberosities along distal half of shaft:
(0) – absent
(1) – present
(Modified from Makovicky and Norell, 2006:144)

330. Ulna, size of olecranon process:
(0) – moderately tall, occupies 32% of less total length of ulna
(1) – high, occupies at least 40% total length of ulna
(Modified from Adams, 1988; Forster, 1990: 104; Makovicky & Norell, 2006:123; Sampson et al., 2010:134; Farke et al., 2011:83)

331. Distal carpals:
(0) – more than two distal carpals
(1) – less than two distal carpals
(Makovicky & Norell, 2006:124)

332. Manus and pes size:
(0) – manus much smaller than pes
(1) – closer to pes in size
(Makovicky & Norell, 2006:125)

333. Manal unguals, shape:
(0) – mediolaterally compressed, taper to distal tip
(1) – dorsoventrally compressed, blunt or rounded distal tip
(Brown, 1914b; Chinnery & Weishampel, 1998:64; Sampson et al, 2010:136; Farke et al., 2011:85)

334. Manal penultimate phalanges, shape:
(0) – length exceeds width
(1) – width exceeds length
(Sampson et al., 2010:137; Farke et al., 2011:86)

335. Manus, relative lengths in digit three:
(0) – articulated phalanges exceed MCIII in length
(1) – articulated phalanges shorter than MCIII in length
(New Character)

336. Manal and pedal phalanges, flexor pits:
(0) – present
(1) – absent
(New Character)

Pelvic Girdle and Limb

337. Ilium, lateral eversion of dorsal margin:
(0) – absent
(1) – present
(Hatcher et al., 1907; Forster 1990: 108 & 109; Chinnery, 2001, 2004; Sampson et al., 2010:138; Farke et al., 2011:87)

338. Ilium, relative lengths of pubic and ischial peduncles:
(0) – pubic and ischial peduncles long, extend well below body of ilium approximately the same distance
(1) – ischial peduncle reduced along ventral aspect, pubic peduncle projects further ventrally than ischial peduncle
(Sampson et al., 2010:139; Farke et al., 2011:88)

339. Pubis, prepubic process:
(0) – short and unexpanded distally
(1) – elongate, distal end greatly expanded dorsoventrally
(Forster 1990:111; Makovicky and Norell, 2006:128; Sampson et al., 2010:140; Farke et al., 2011:89)

340. Pubis, position and length of postpubic rod:
(0) – relatively long but extends past ischial peduncle of ilium, arises ventral to acetabulum and lies along ventral and ventromedial margin of ischium
(1) – very abbreviated, terminates at level of ischial peduncle, arises medial to acetabulum and passes entirely medial to ischium
(Forster, 1990:110; Makovicky and Norell, 2006:127; Sampson et al., 2010:141; Farke et al., 2011:90)

341. Pubis and ischium, morphology of contributions to acetabulum:
(0) – pubic acetabular surface faces posteriolaterally, pubis and pubic process of ischium contribute equally to ventral margin of acetabulum
(1) – pubic acetabular surface faces laterally and forms a partial medial wall to the acetabulum, pubic process of ischium elongate and meets pubis close to anterior margin of acetabulum, ventral portion of pubic acetabular surface lies medial to pubic ramus of ischium
(Sampson et al., 2010:142; Farke et al., 2011:91)

342. Pubis, cross-sectional shape of postpubic shaft:
(0) – round
(1) – mediolaterally flattened, bladelike
(Modified from Makovicky and Norell, 2006:126)

343. Ischium, cross-sectional shape of shaft:
(0) – thick and ovoid
(1) – laterally compressed and bladelike, tapered dorsally
(Forster 1990:112; Sampson et al., 2010:143; Farke et al., 2011:92)

344. Ischium, orientation of shaft:
(0) – nearly straight or slightly decurved
(1) – broadly and continuously curved
(Brown & Schlaikjer, 1940a; Forster 1990:113; Makovicky and Norell, 2006:129; Sampson et al., 2010:144; Farke et al., 2011:93)

345. Femur, morphology of greater and lesser trochanters:
(0) – trochanters distinct and located below the level of the femoral head
(1) – trochanters coalesced and level with the femoral head
(Brown & Schlaikjer, 1940a; Dodson et al., 2004:72; Sampson et al., 2010:145; Farke et al., 2011:94)

346. Femur, size of fourth trochanter:
(0) – large and pendant
(1) – small, reduced to low prominence
(Brown & Schlaikjer, 1940a; Sereno 1999:154; Makovicky and Norell, 2006:130; Sampson et al., 2010:146; Farke et al., 2011:95)

347. Femur-tibia proportion:
(0) – tibia longer than, or equal to femur length
(1) – femur longer than tibia
(Brown & Schlaikjer, 1940a; Forster 1990:103; Makovicky and Norell, 2006: 131; Sampson et al., 2010:147; Farke et al., 2011:96)

348. Pes, metatarsal proportions:
(0) – length of MT I 80% the length of MT II
(1) – MT I reduced less than 70% the length of MT II

(Modified from Sampson et al., 2010:148; Farke et al., 2011:97)

349. Pes, shape:

(0) – gracile with long, constricted metatarsus, elongate phalanges

(1) – short and uncompressed, all phalanges wider than long

(Modified from Makovicky and Norell, 2006:132)

350. Pedal unguals, shape:

(0) – pointed

(1) – rounded, hooflike

(Modified from Makovicky and Norell, 2006:133)

References

Adams DA. (1988) Structure and function of the ceratopsian forelimb. *Ph.D. Dissertation, University of California, Berkeley*, 379 pp

Arbour, V. M.; Burns, M. E.; Sissons, R. L. (2009). A redescription of the ankylosaurid dinosaur *Dyoplosaurus acutosquameus* Parks, 1924 (Ornithischia: Ankylosauria) and a revision of the genus. *Journal of Vertebrate Paleontology* **29 (4):** 1117–1135

Bell MA and Lloyd GT (2014). strap: Stratigraphic Tree Analysis for Palaeontology. R package version 1.4. https://CRAN.R-project.org/package=strap

Brown B. (1914) *Leptoceratops*, a new genus of Ceratopsia from the Edmonton Cretaceous of Alberta. *Bull. Am. Mus. Nat. Hist*. **33**, 567–580

Brown B, Schlaikjer EM. (1940) The structure and relationships of *Protoceratops*. *Ann. N. Y. Acad. Sci*. **40**, 133–266. (doi:10.1111/j.2164-0947.1940.tb00068.x)

Brown, C.M. and Henderson, D.M. (2015). A new horned dinosaur reveals convergent evolution in cranial ornamentation in Ceratopsidae. *Current Biology*. **25**; 1641 – 1648

Changfu, Z., Keqin, G., Fox, RC.; Shuihua, C. (2006). A new species of Psittacosaurus (Dinosauria: Ceratopsia) from the Early Cretaceous Yixian Formation, Liaoning, China. *Palaeoworld* **15:** 100–114

Chinnery, BJ, & Weishampel, DB (1998). Montanoceratops cerorhynchus (Dinosauria: Ceratopsia) and relationships among basal neoceratopsians. *Journal of Vertebrate Paleontology*, **18**; 569-585

Chinnery B. (2004) Description of *Prenoceratops pieganensis* gen. et. sp. nov. (Dinosauria: Neoceratopsia) from the Two Medicine Formation of Montana. *J. Vertebr. Paleontol*. **24**, 572–590. (doi:10.1671/0272-4634(2004)024[0572:DOPPGE]2.0.CO;2)

Currie, P.J., Langston, W., and Tanke, D.H. (2008). A new species of Pachyrhinosaurus (Dinosauria, Ceratopsidae) from the Upper Cretaceous of Alberta, Canada. pp. 1-108. In: Currie, P.J., Langston, W., and Tanke, D.H. 2008. *A New Horned Dinosaur from an Upper Cretaceous Bone Bed in Alberta*. NRC Research Press, Ottawa, Ontario, Canada. 144 pp

Dashzeveg, D., L. Dingus, D. B. Loope, C. C. Swisher, T. Dulam, and M. R. Sweeney (2005). New stratigraphic subdivision, depositional environment, and age estimate for the Upper Cretaceous Djadokhta Formation, Southern Ulan Nur Basin, Mongolia. *American Museum Novitates* **3498**:1–31

Dodson, P. (1986). *Avaceratops lammersi*: a new ceratopsid from the Judith River Formation of Montana. *Proceedings of the Academy of Natural Sciences of Philadelphia*. **138** (2): 305–317

Dodson P, Forster CA, Sampson SD. (2004) Ceratopsidae. In *The Dinosauria* (eds DB Weishampel, P Dodson, H Osmólska), pp. 494–513. Berkeley: University of California Press.

Evans DC, Ryan MJ. (2015) Cranial anatomy of *Wendiceratops pinhornensis* gen. et sp. nov., a centrosaurine ceratopsid (Dinosauria: Ornithischia) from the Oldman Formation (Campanian), Alberta, Canada, and the evolution of ceratopsid nasal ornamentation. *PLoS ONE* **10**, e0130007. (doi:10.1371/journal.pone.0130007)

Farke AA. (2006) Cranial osteology and phylogenetic relationships of the chasmosaurine ceratopsid *Torosaurus latus*. In *Horns and Beaks: Ceratopsian and Ornithopod Dinosaurs* (ed K Carpenter), pp. 235–257. Bloomington: Indiana University Press

Farke, A.A. (2007). Cranial osteology and phylogenetic relationships of the chasmosaurine ceratopsid *Torosaurus latus.* In Carpenter, K. (ed.). *Horns and Beaks.* Indiana University Press, Bloomington and Indiana.

Farke AA (2011) Anatomy and Taxonomic Status of the Chasmosaurine Ceratopsid *Nedoceratops hatcheri* from the Upper Cretaceous Lance Formation of Wyoming, U.S.A.. *PLoS ONE* **6(1)**: e16196. doi:10.1371/journal.pone.0016196

Farke, AA., Ryan, M.J., Barrett, P.M., Tanke, D.H., Braman, D.M., Loewen, M.A. and Graham, M.R. (2011). A new centrosaurine from the Late Cretaceous of Alberta, Canada, and the evolution of parietal ornamentation in horned dinosaurs. *Acta Palaeontologica Polonica*. **56** (4): 691–702.

Farke, AA. Maxwell, WD., Cifelli, RL., Wedel, MJ. (2014). A Ceratopsian Dinosaur from the Lower Cretaceous of Western North America, and the Biogeography of Neoceratopsia. *PLoS ONE*. **9 (12)**: e112055

Fiorello, A.R. and Tykoski, R.S. (2011). A new Maastrichtian species of the Centrosaurine ceratopsid *Pachyrhinosaurus* from the north slope of Alaska. *Acta Palaeontologica. Pol.* **57**; 561 – 573

Forster CA. (1990) The cranial morphology and systematics of *Triceratops* with a preliminary analysis of ceratopsian phylogeny. *Ph.D. Dissertation, University of Pennsylvania*, 227 pp

Forster CA, Sereno PC, Evans TW, Rowe T. (1993) A complete skull of *Chasmosaurus mariscalensis* (Dinosauria: Ceratopsidae) from the Aguja Formation (Late Campanian) of west Texas. *J. Vertebr. Paleontol*. **13**, 161–170.

Gao, K., and Cheng, Z. (1999). A new lizard from the Lower Cretaceous of Shandong, China. *Journal of Vertebrate Paleontology* **19**: 456–465

Gregory WK, Mook CC. (1925) On *Protoceratops*, a primitive ceratopsian dinosaur from the Lower Cretaceous of Mongolia. *Am. Mus. Novit*. **156**, 1–9.

Han, F., Forster, C.A., Clark, J.M. and Xu, X. (2015). A new taxon of basal ceratopsian from China and the early evolution of ceratopsian. *PLOSone.* DOI:10.1371/journal.pone.0143369

Hatcher JB, Marsh OC, Lull RS. (1907). *The Ceratopsia*. *U. S. Geol. Surv. Monogr*. **49**, 1–300

Holmes RB, Forster C, Ryan M, Shephard KM. (2001) A new species of *Chasmosaurus* (Dinosauria: Ceratopsia) from the Dinosaur Park Formation of southern Alberta. *Can. J. Earth Sci*. **38**, 1423–1438

Kirkland, J.I. and DeBlieux, D.D. (2010). New basal centrosaurine ceratopsian skulls from the Wahweap Formation (Middle Campanian), Grand Staircase–Escalante National Monument, southern Utah, In: Ryan, M.J., Chinnery-Allgeier, B.J., and Eberth, D.A. (eds.) *New Perspectives on Horned Dinosaurs: The Royal Tyrrell Museum Ceratopsian Symposium*. Bloomington, Indiana University Press, pp. 117–140

Lambe LM. (1915) On *Eoceratops canadensis*, gen. nov., with remarks on other genera of Cretaceous horned dinosaurs. *Can. Geol. Surv. Mus. Bull*. **12**, 1–49

Lambert, O, Godefroit, P, Li, H, Shang, C.-Y. & Dong,Z.-M. (2001). A new species of *Protoceratops* (Dinosauria, Neoceratopsia) from the Late Cretaceous of Inner Mongolia (P. R. China). *Bulletin de l'Institut Royal des Sciences Naturelles de Belgique, Science de la Terre*: 5–28

Lee, Y-N., Ryan, M.J., Kobayashi, Y. (2011). The first ceratopsian dinosaur from South Korea. *Naturwissenschaften* **98 (1)**: 39–49

Lehman TM. (1989) *Chasmosaurus mariscalensis*, sp. nov., a new ceratopsian dinosaur from Texas. *J. Vertebr. Paleontol*. **9**, 137–162

Lehman TM. (1990) The ceratopsian subfamily Chasmosaurinae: sexual dimorphism and systematics. In *Dinosaur Systematics: Approaches and Perspectives* (eds K Carpenter, PJ Currie), pp. 211–229. Cambridge: Cambridge University Press

Lehman TM. (1996) A horned dinosaur from the El Picacho Formation of West Texas, and review of ceratopsian dinosaurs from the American Southwest. *J. Paleontol*. **70**, 494–508

Liyong, J. Jun, C., Shuqin, Z. and Godefroit, P. (2009). A New Basal Neoceratopsian Dinosaur from the Middle Cretaceous of Jilin Province, China. *Acta Geologica Sinica.* **83 (2)**: 200. doi:10.1111/j.1755-6724.2009.00023.x.

Longrich, N.R. (2010). *Mojoceratops perifania,* a new chasmosaurine ceratopsid from the late Campanian of Western Canada. *Journal of Paleontology.* **84;** 681 – 694

Longrich, N.R. (2011). *Titanoceratops ouranos*, a giant horned dinosaur from the late Campanian of New Mexico. *Cretaceous Research.* **32**; 264 – 276

Lull RS. (1933) A revision of the Ceratopsia or horned dinosaurs. *Mem. Peabody Mus. Nat. Hist*. **3**, 1–175

Lund, E.K. (2010). *Nasutuceratops titusi*, a new basal centrosaurine dinosaur (Ornithischia: Ceratopsidae) from the Upper Cretaceous Kaiparowits Formation, Southern Utah. *Department of Geology and Geophysics University of Utah*: 172 pp

Maidment SCR, Barrett PM. (2011) A new specimen of *Chasmosaurus belli* (Ornithischia: Ceratopsidae), a revision of the genus, and the utility of postcrania in the taxonomy and systematics of ceratopsid dinosaurs. *Zootaxa* **2963**, 1–47. (doi:10.11646/zootaxa.2963.1.1)

Makovicky PJ. (2001) A *Montanoceratops cerorhynchus* (Dinosauria: Ceratopsia) braincase from the Horseshoe Canyon Formation of Alberta. In *Mesozoic Vertebrate Life* (eds DH Tanke, K Carpenter), pp. 243–262. Bloomington, Indiana: Indiana University Press

Makovicky P, Norell MA. (2006) *Yamaceratops dorngobiensis*, a new primitive ceratopsian (Dinosauria: Ornithischia) from the Cretaceous of Mongolia. *Am. Mus. Novit*. **3530**, 1–42. (doi:10.1206/0003-0082(2006)3530[1:YDANPC]2.0.CO;2)

Mallon JC, Ott CJ, Larson PL, Iuliano EM, Evans DC (2016) *Spiclypeus shipporum* gen. et sp. nov., a Boldly Audacious New Chasmosaurine Ceratopsid (Dinosauria: Ornithischia) from the Judith River Formation (Upper Cretaceous: Campanian) of Montana, USA. *PLoS ONE*. **11(5):** e0154218.

Marsh OC. (1892) The skull of *Torosaurus. Am. J. Sci. 43*, 81–84

Maryańska, T and H. Osmólska (1975). Protoceratopsidae (Dinosauria) of Asia. *Palaeontologia Polonica.* **33**: 133-181

McDonald, A.T., and Horner, J.R. (2010). New material of *"Styracosaurus" ovatus* from the Two Medicine Formation of Montana. In: Ryan, M.J., Chinnery-Allgeier, B.J., and Eberth, D.A. (eds.) *New Perspectives on Horned Dinosaurs: The Royal Tyrrell Museum Ceratopsian Symposium*. Bloomington, Indiana University Press. Pp 156 – 168

Osi, A., Butler, RJ., and Weishampel, DB. (2010). A Late Cretaceous ceratopsian dinosaur from Europe with Asian affinities. *Nature.* **465:** 466 – 468

Osmólska H. (1986) Structure of nasal and oral cavities in the protoceratopsid dinosaurs (Ceratopsia, Ornithischia). *Acta Palaeontol. Pol*. **31**, 145–157

Ostrom JH. (1964) A functional analysis of jaw mechanics in the dinosaur *Triceratops. Postilla* **88**, 1–35

Ostrom JH, Wellnhofer P. (1986) The Munich specimen of *Triceratops* with a revision of the genus. *Zitteliana* **14**, 111–158.

Ott, C.J. (2007). Cranial anatomy and biogeography of the first *Leptoceratops gracilis* (Dinosauria: Ornithischia) specimens from the Hell Creek Formation, southeast Montana. In Carpenter, K. (ed.). *Horns and Beaks.* Indiana University Press, Bloomington and Indiana.

Renne; et al. (2013). Time Scales of Critical Events Around the Cretaceous-Paleogene Boundary. *Science.* **339**: 684–7

Roberts, E. M., S. D. Sampson, A. Deino, S. Bowring, and R. Buchwaldt (2013). The Kaiparowits Formation: a remarkable record of Late Cretaceous terrestrial environments, ecosystems and evolution in Western North America; pp. 85–106 in A. L. Titus and M. A. Loewen (eds.), *At the Top of the Grand Staircase: The Late Cretaceous of Southern Utah*. Indiana University Press, Bloomington, Indiana.

Ryan, M.J. and Russell, A.P. (2005). A new centrosaurine dinosaur from the Oldman Formation of Alberta and its implications for centrosaurine taxonomy and systematics. *Can. J. Earth Sci.* **42**: 1369 – 1387

Ryan, M.J., Eberth, D.A., Brinkman, D.B., Currie, P.J. and Tanke, D.H. (2010). A new *Pachyrhinosaurus*-like ceratopsid from the Upper Dinosaur Park Formation (Late Campanian) of southern Alberta, Canada. In: Michael J. Ryan, Brenda J. Chinnery-Allgeier, and David A. Eberth (eds), *New Perspectives on Horned Dinosaurs: The Royal Tyrrell Museum Ceratopsian Symposium*, Indiana University Press, 141 pp

Ryan, M.J., Evans, D.C., Currie, P.J., Brown, C.M., Brinkman, D. (2012). New leptoceratopsids from the Upper Cretaceous of Alberta, Canada. *Cretaceous Research*. **35**: 69–80.

Ryan, M. J.; Evans, D. C.; Currie, P. J.; Loewen, M. A. (2014). A new chasmosaurine from northern Laramidia expands frill disparity in ceratopsid dinosaurs. *Naturwissenschaften.* **101**: 505–512

Sampson, S.D. (1995). Two new horned dinosaurs from the Upper Cretaceous Two Medicine Formation of Montana; with a phylogenetic analysis of the Centrosaurinae (Ornithischia: Ceratopsidae). *Journal of Vertebrate Paleontology*. **15(4)**: 743-760

Sampson SD, Ryan MJ, Tanke DH. (1997) Craniofacial ontogeny in centrosaurine dinosaurs (Ornithischia: Ceratopsidae): taxonomic and behavioral implications. *Zool. J. Linn. Soc*. **121**, 293–337. (doi:10.1111/j.1096-3642.1997.tb00340.x)

Sampson, S.D., and Loewen, M.A. (2010). Unravelling a radiation: a review of the diversity, stratigraphic distribution, biogeography and evolution of horned dinosaurs (Ornithischia: Ceratopsidae). In: Michael J. Ryan, Brenda J. Chinnery-Allgeier, and David A. Eberth (eds), *New Perspectives on Horned Dinosaurs: The Royal Tyrrell Museum Ceratopsian Symposium*, Indiana University Press, pp 405

Sampson SD, Loewen MA, Farke AA, Roberts EM, Forster CA, Smith JA, Titus AL. (2010) New horned dinosaurs from Utah provide evidence for intracontinental dinosaur endemism. *PLOS ONE* **5**, e12292

Scanella, J.B., Fowler, D.W., Goodwin, M.B., and Horner, J.R. (2014). Evolutionary trends in *Triceratops* from the Hell Creek Formation, Montana. *PNAS.* **111;** 10245 – 10250

Sereno PC. (1999) The evolution of dinosaurs. *Science* **284**, 2137–2147

Sereno, P.C. (2010). Taxonomy, cranial morphology and relationships of parrot-beaked dinosaurs (Ceratopsia: *Psittacosaurus*). In: Michael J. Ryan, Brenda J. Chinnery-Allgeier, and David A. Eberth (eds), *New Perspectives on Horned Dinosaurs: The Royal Tyrrell Museum Ceratopsian Symposium*, Indiana University Press, pp 21.

Shuvalov, V.F. (2000) The Cretaceous stratigraphy and paleobiologeography of Mongolia. In: Benson, M.J., Shishkin, M.A., Unwin, D.M. and Kurochkin, E.N. *The Age of Dinosaurs in Russia and Mongolia.* Cambridge University Press, Cambridge.

Sues, H.-D., and Averianov, A. (2009). *Turanoceratops tardabilis*—the first ceratopsid dinosaur from Asia. *Naturwissenschaften*. **96**: 645 – 652

Sullivan, R.M., Boere, A.C., and Lucas, S.G. (2005). Redescription of the ceratopsid dinosaur *Torosaurus utahensis* (Gilmore, 1946) and a revision of the genus. *J. Paleont.* **79:** 564 – 582

Swisher, C.C., Wang, Y, Wang, X, Xu, X., Wang, Y. (1999). Cretaceous age for the feathered dinosaurs of Liaoning, China. *Nature.* **400:** 58–61

Wick, S.L. and Lehman, T.M. (2013). A new ceratopsian dinosaur from the Javelina Formation (Maastrchtian) of West Texas and implications for chasmosaurine phylogeny. *Naturwissenschaften.* **100**; 667 – 682

Wolfe, D.G., Kirkland, J.I., Smith, D., Poole, K., Chinnery-Allgeier, B., and McDonald, A. (2010). *Zuniceratops christopheri*: The North American ceratopsid sister taxon reconstructed on the basis of new data. In: Ryan, M.J., Chinnery-Allgeier, B.J. and Eberth, D.A. (eds.) *New Perspectives on Horned Dinosaurs.* Indiana University Press, Bloomington and Indianapolis

Xu, X., Makovicky, P.J., Wang, X, Norell, M.A. and You, H. (2002). A ceratopsian dinosaur from China and the early evolution of Ceratopsia. *Nature*. **416** (6878): 314–317

Xu, X., Forster, C.A., Clark, J.M., and Mo, J. (2006). A basal ceratopsian with transitional features from the Late Jurassic of northwestern China. *Proc. R. Soc. B.* doi:10.1098/rspb.2006.3566

Xu, X., Wang, K., Zhao, X. & Li, D. (2010). First ceratopsid dinosaur from China and its biogeographical implications. *Chinese Science Bulletin* **55 (16):** 1631–1635

You H.-L. & Dong, Z. (2003). A new protoceratopsid (Dinosauria: Neoceratopsia) from the Late Cretaceous of Inner Mongolia, China. *Acta Geologica Sinica* (English edition) **77** (3): 299–303

You, H., Li, D., Ji, Q., Lamanna, M. and Dodson, P. (2005). On a new genus of basal Neoceratopsian dinosaur from the Early Cretaceous of Gansu Province, China. *Acta Geologica Sinica.* **79 (5)**; 593-597Zhao, X., Cheng, Z., & Xu, X. (1999). The earliest ceratopsian from the Tuchengzi Formation of Liaoning, China. *Journal of Vertebrate Paleontology*. **19(4)**: 681-691

You, H., Tanoue, K and Dodson, P (2008). New data on cranial anatomy of the ceratopsian dinosaur Psittacosaurus major. *Acta Palaeontologica Polonica* **53 (2):** 183–196

Zhao, X., Zhengwu, C., Xu, X. and Makovicky, P.J. (2006). A new ceratopsian from the Upper Jurassic Houcheng Formation of Hebei, China. *Acta Geologica Sinica* **80:**467–473

Zheng, W., Jin, X., & Xu, X. (2015). A psittacosaurid-like basal neoceratopsian from the Upper Cretaceous of central China and its implications for basal ceratopsian evolution. *Scientific Reports*. **5**. 14190: 1-9; doi:10.1038/srep14190
